# Selectively fluorinated cyclohexane building blocks: Derivatives of carbonylated all-*cis-*3-phenyl-1,2,4,5-tetrafluorocyclohexane

**DOI:** 10.3762/bjoc.11.287

**Published:** 2015-12-21

**Authors:** Mohammed Salah Ayoup, David B Cordes, Alexandra M Z Slawin, David O'Hagan

**Affiliations:** 1 School of Chemistry, University of St Andrews, North Haugh, St Andrews, KY16 9ST, UK; 2 Department of Chemistry, Faculty of Science, P.B 426 Ibrahimia, Alexandria University, Egypt

**Keywords:** cyclohexane carbonylation, fluorine containing building blocks: organofluorine chemistry

## Abstract

Palladium catalysed carbonylation reactions using the *meta*- and *para*-iodo derivatives of all-*cis-*3-phenyl-1,2,4,5-tetrafluorocyclohexane (**4**) are illustrated as the start point for a variety of functional group interconversions. The resultant benzaldehyde and benzoic acids offer novel building blocks for further derivatisation and facilitate the incorporation of the facially polarised all-*cis-*1,2,4,5-tetrafluorocyclohexane motif into more advanced molecular scaffolds.

## Introduction

**Figure 1:**
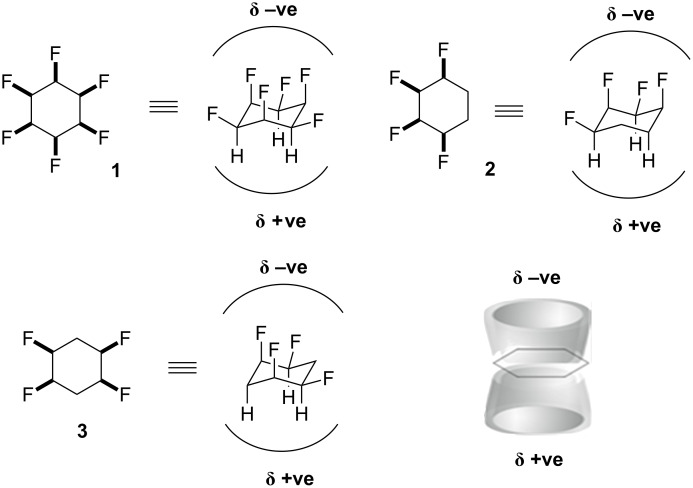
All *cis*-hexafluoro- **1** and tetrafluorocyclohexanes **2** and **3** result in facially polarised ring motifs with an electrostatically negative (δ –ve) fluorine face and an electrostatically positive (δ +ve) hydrogen face.

Selectively fluorinated building blocks have proven invaluable in drug discovery [1] and agrochemical research programmes [2]. Most such building blocks possess aryl/heteroaryl-F, aryl/heteroaryl-(X)CF_3_ or variants thereof, whereas selectively fluorinated aliphatics are much rarer. We have had a focus on introducing selectively fluorinated cyclohexane rings, to complement aromatic building blocks. In this context we have recently prepared the all-*cis*-tetrafluorocyclohexanes **2** and **3** [3–4] and also all-*cis-*hexafluorocyclohexane (**1**) [5] as shown in Figure 1. In the case of the hexafluorocyclohexane, the ring maintains a chair conformation and therefore there are three C–F bonds orientated triaxial, on one face of the ring. This gives rise to a large dipole moment (6.2 D) and a molecule which is among the most polar aliphatics known in organic chemistry. The all-*cis-*tetrafluorocyclohexanes **2** and **3** are also facially polarised because in the chair conformation, they always have two 1,3-diaxial C–F bonds on one face of the ring, and this results in ring systems also with large dipole moments (4.9 D for **2** and 5.2 D for **3**). The nature of these cyclohexanes, where the two faces are oppositely polarised, presents a unique property for use in pharmaceuticals and agrochemicals discovery programmes.

Compounds **1–3** are unfunctionalised and not amenable to derivatisation, and therefore we started to explore the chemistry of all-*cis*-3-phenyl-1,2,4,5-tetrafluorocyclohexane (**4**) [6], to access derivatives carrying this ring motif. In that context it has been demonstrated that **4** can be elaborated in a relatively straightforward manner by mainstream reactions of electrophilic aromatic substitution [7]. This extended to the synthesis of cyclohexane substituted (*S*)-L-phenylalanines with orthogonal protecting groups suitable for their incorporation into peptides [8]. In this paper it is demonstrated that palladium mediated carbonylation of the aryl iodinated derivatives **5–7** forms the basis of a diversity of new products which may prove attractive as building blocks for structure activity studies in bioactive research projects.

## Results and Discussion

**Scheme 1:**
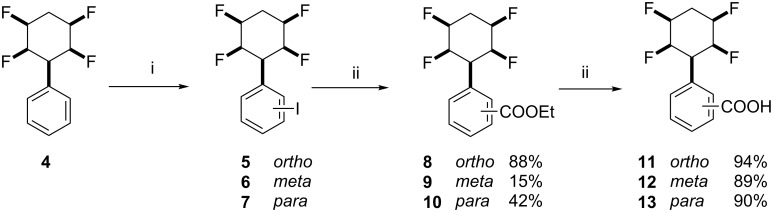
Preparation of benzoic acids **11**–**13**; i. HIO_4_·2H_2_O (50%), AcOH, H_2_SO_4_, I_2_, H_2_O, 70 °C 24 h, 92%.; ii. Pd(OAc)_2_, Ph_3_P,Et_3_N, EtOH, CO (1 atm), 80 °C, 16 h. iii. HCl (6 M), 1,4-dioxane, 70 °C, 24 h or TFA/H_2_O (9:1), 100 °C, 24 h.

A key reaction for this programme involved the carbonylation of aryl iodides **5**, **6** and **7** which are derived by HIO_4_ treatment of all-*cis*-3-phenyl-1,2,4,5-tetrafluorocyclohexane (**4**). The *ortho* isomer **5** was separated from the *meta* and *para* isomers **6** and **7**, the latter of which were recovered as a mixture as previously reported [8]. Palladium catalysed carboxylation [9] was explored with aryl iodides **5** or **6**/**7** as a mixture, and these gave the corresponding ethyl esters **8** and **9**/**10**, respectively. The *meta* and *para* esters **9** and **10** were easily separated by chromatography. Hydrolysis of esters **8**–**10** using trifluoroacetic acid (TFA) or 6 M HCl in dioxane [10] gave the corresponding benzoic acids **11**, **12**, and **13** as illustrated in Scheme 1.

**Scheme 2:**
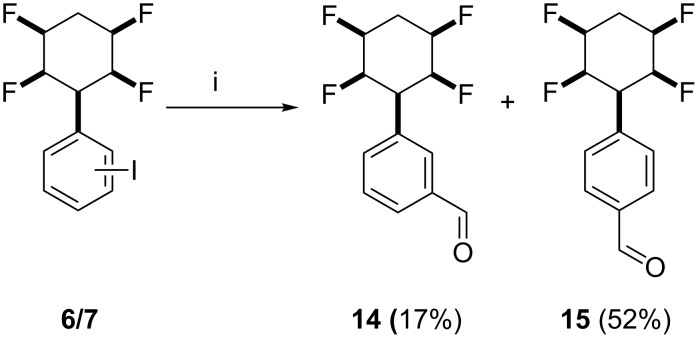
Synthesis of benzaldehyde derivatives **14** and **15**: i. Pd(PPh_3_)_4_, Bu_3_SnH, THF, CO (1 atm), 50 °C, 2–3 h, 70%.

**Figure 2:**
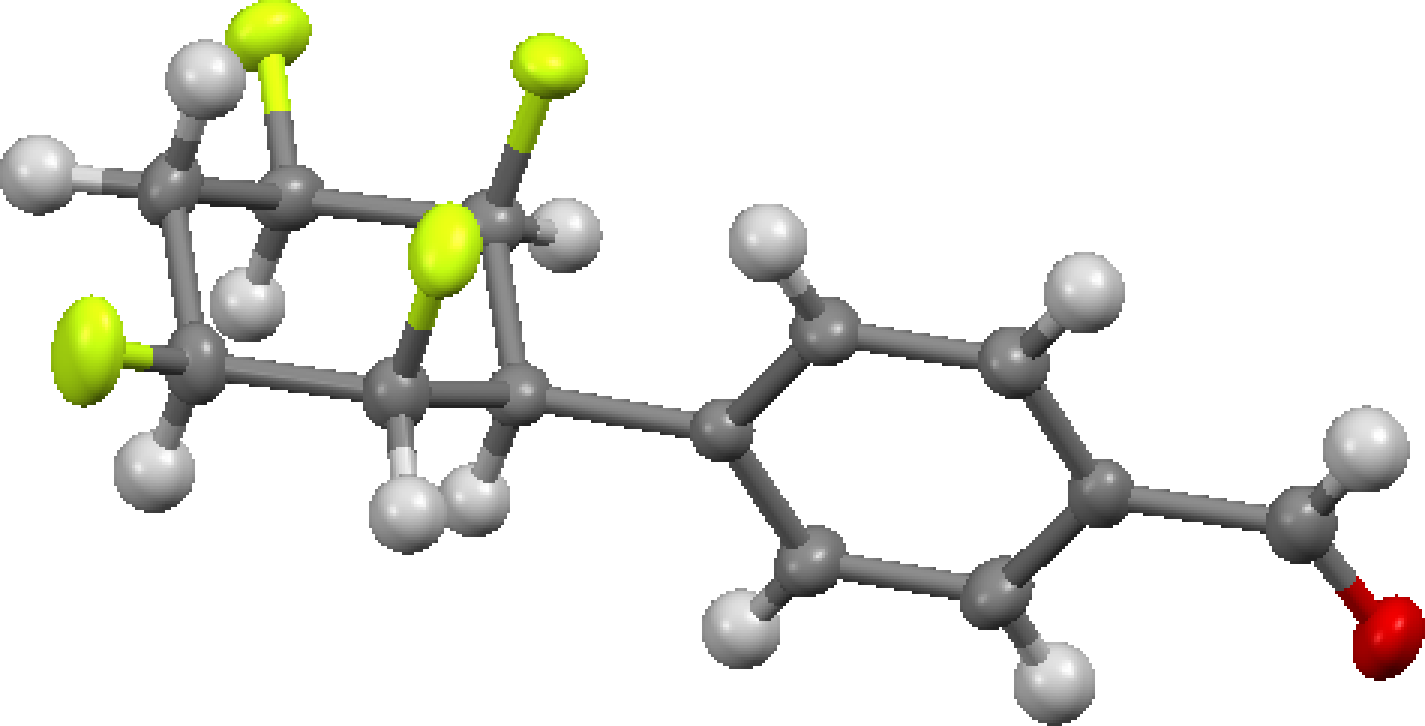
X-ray structure of aldehyde **15**. CCDC number 1432193.

Direct palladium catalysed formylation of aryl iodides **6**/**7** as a mixture was explored using Pd(PPh_3_)_4_, Bu_4_SnH and CO [11]. This generated benzaldehyde derivatives **14** and **15** in a 1:3 ratio, respectively, which could be separated by column chromatography. The reaction is illustrated in Scheme 2. The structure of aldehyde **15** was confirmed by X-ray structure analysis and is shown in Figure 2.

**Scheme 3:**
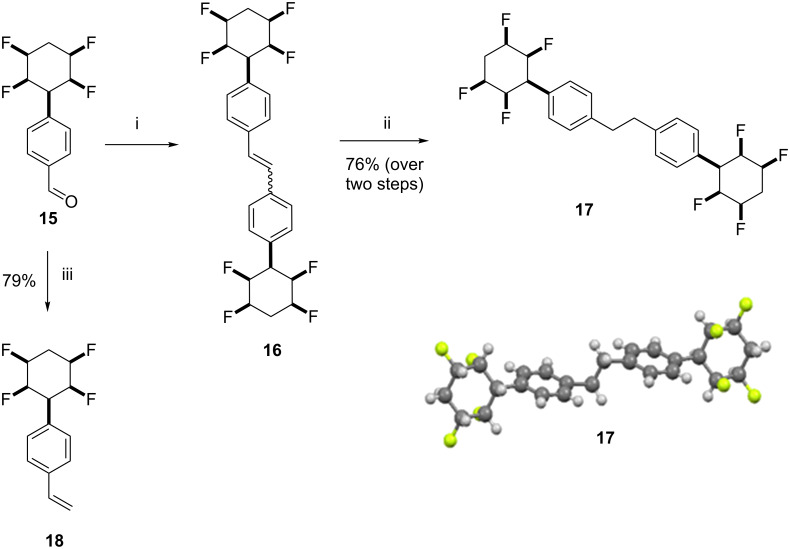
Olefination reactions of **15** and the X-ray structure of **17** (CCDC number 1432194): i. Zn, TiCl_4_, THF, 0 °C to reflux, 12 h; ii. Pd/C, H_2_, EtOAc, 20 °C, 16 h, 76% over 2 steps. iii. Zn, CH_2_I_2_, TiCl_4_, THF, 0 °C to 20 °C, 12 h; 79%.

Transformations of benzaldehyde **15** were explored. For example McMurry coupling [12] of **15** gave a mixture of stilbene isomers **16**. Direct hydrogenation of the mixture afford dihydrostilbene **17** as a crystalline solid, a structure which was confirmed by X-ray crystallography and is shown in the inset in Scheme 3. Aldehyde **15** could also be converted to styrene **18** in good yield using the Simmons–Smith method [13] as illustrated in Scheme 3.

**Scheme 4:**
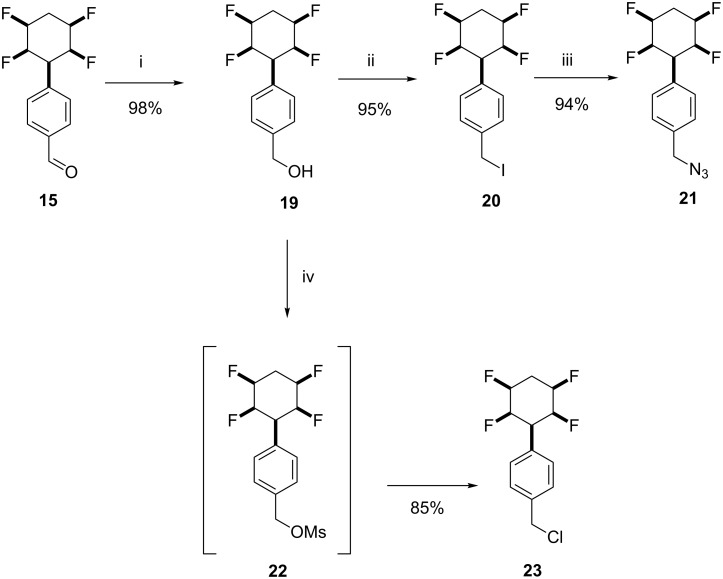
Reactions from aldehyde **15**: i. NaBH_4_, THF, 20 °C, 1 h, 98%.; ii. HI (57%), CHCl_3_, 30 h, 95%; iii. Bu_4_NN_3_, acetone/H_2_O (4:1), 20 °C, 3 h, 94%; iv. MsCl, Et_3_N, DCM, −78 °C to 20 °C, 85%.

For further elaboration, aldehyde **15** was reduced with NaBH_4_ in good yield to generate the corresponding benzyl alcohol **19** [14]. Iodination of **19** to generate **20** with HI in chloroform [15] proved superior (95% yield) to the more classical Appel protocol which gave low yields in our hands. Nucleophilic substitution with the resultant benzyl iodide **20** using azide gave the corresponding benzyl azide **21** in good yield [16]. Chlorination to generate **23** was accomplished by treatment with mesyl chloride/Et_3_N in a one pot protocol, presumably via mesylate **22**, as illustrated in Scheme 4 [17].

**Scheme 5:**
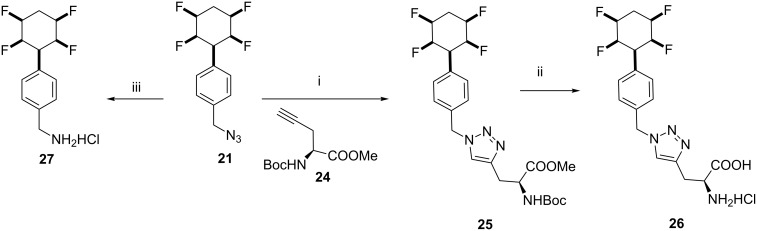
Reactions of benzyl azide **21**; i. **24**, Cu(OAc)_2_, Na ascorbate, *t*-BuOH, H_2_O, 20 °C, 16 h, 72%; ii. HCl (6 M), 1,4-dioxane, 80 °C, 48 h, 96%; iii. Ph_3_P, THF/H_2_O (10:1), then HCl, rt, 1 h, 74%.

Reduction of benzyl azide **21** to generate amine **27** as its hydrochloride salt was achieved by a Staudinger reduction in good yield [18]. Azide **21** was also amenable to a ‘click’ reaction, in this case with the acetylenic protected amino acid **24** [19]. This gave the 1,2,3-triazol linked adduct **25** which could be fully deprotected to generate the free amino acid hydrochloride **26** as illustrated in Scheme 5.

**Scheme 6:**
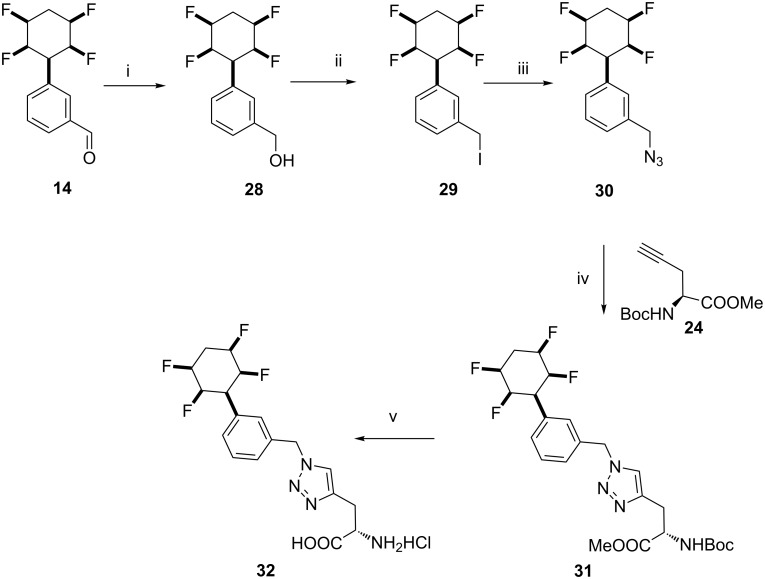
Reactions of aldehyde **14**: i. NaBH_4_, THF, rt, 1 h, 98%.; ii. HI (57%), CHCl_3_, 30 h, 94%; iii. Bu_4_NN_3_, acetone/H_2_O (4:1), rt, 3 h, 91%; iv. **24**, Cu(OAc)_2_, Na ascorbate, *t*-BuOH, H_2_O, rt, 12 h, 81%; v. HCl (6 M), 1,4-dioxane, 70 °C, 48 h, 94%.

The reactions described above were equally amenable to the *meta*-benzaldehyde **14**, as illustrated in Scheme 6.

## Conclusion

In summary it is demonstrated that the all-*cis-*1,2,4,5-tetrafluorocyclohexane motif has been incorporated in a range of products prepared from aryl iodides **5–7**. These derivatives derive from aryl carboxylation or carbonylation, and complement those that can be prepared directly by electrophilic aromatic substitution of phenyl derivative **4**. This chemistry should more readily facilitate the exploration of the properties and potential of the all-*cis-*1,2,4,5-tetrafluorocyclohexane motif in a diversity of research programmes.

## Supporting Information

File 1 Experimental part.
