# Supplementary material for: Selectively fluorinated cyclohexane building blocks: Derivatives of carbonylated all-cis-3-phenyl-1,2,4,5-tetrafluorocyclohexane
Source: Beilstein J Org Chem. 2015 Dec 21;11:2671–6. doi: 10.3762/bjoc.11.287 (PMC4734450; doi:10.3762/bjoc.11.287)

Supporting Information

for

Selectively fluorinated cyclohexane building blocks: Derivatives of carbonylated all-*cis*-3-phenyl-1,2,4,5-tetrafluorocyclohexane

Mohammed Salah Ayoup[1,2], David B. Cordes[1], Alexandra M. Z. Slawin[1] and David O'Hagan[1]*

Address: [1]School of Chemistry, University of St Andrews, North Haugh, St Andrews, KY16 9ST, UK and [2]Department of Chemistry, Faculty of Science, P.B 426 Ibrahimia, Alexandria University, Egypt

Email: David O'Hagan – do1@st-andrews.ac.uk

*Corresponding author

Table of Content

1- General experimental notes…………………………S2
2- Procedure and analytical data ………………..S3–S13
3- NMR spectra and mass spectra……………..S14–S35

1. General experimental

All reactions were carried out in oven-dried glassware under argon. Petrol refers to the petroleum ether fraction 40–60 °C. All chemicals were used as supplied. All NMR spectra were recorded using a Bruker Avance III 500, Bruker Avance II 400 or Bruker Avance 300 spectrometers. The deuterated solvent was used as an internal deuterium lock. ^{13}C NMR spectra were recorded using the UDEFT pulse sequence and broadband proton decoupling at either 75, 100 or 126 MHz. ^{19}F NMR spectra were recorded at 282, 376 or 470 MHz. All chemical shifts, δ, are stated in units of parts per million (ppm), relative to a standard, for ^{1}H NMR and ^{13}C NMR the reference point is TMS (δ_H and δ_C: 0.00 ppm). For ^{19}F NMR the external reference used is CCl_3F (δ_F: 0.00 ppm). Melting points were determined using a Griffin MPA350 or an Electrothermal 9100 melting point apparatus and are uncorrected. High and low resolution mass spectra were obtained by atmospheric pressure chemical ionisation (APCI), electrospray ionization (ESI) and electron ionisation (EI). ESI-MS spectra were recorded on a Waters Micromass LCT spectrometer in positive mode or negative mode. EI-MS spectra were recorded on a Thermo Excalibur Orbitrap spectrometer. Values are reported as a ratio of mass to charge (*m*/*z*) in Daltons.

Experimental

Ethyl 3-(all *cis*-2,3,5,6-tetrafluorocyclohexyl)benzoate (9), ethyl 4-(all *cis*-2,3,5,6-tetrafluorocyclohexyl)benzoate (10)

$Pd(OAc)_2$ (7 mg, 1 mol %) was added to a solution of aryl iodide **6**/**7** (100 mg, 0.279 mmol), triphenylphosphine (14 mg, 2 mol %) and Et_3N (0.07 mL, 0.56 mmol) in ethanol (5 mL) in a flame dried round flask. The flask was evacuated and fixed with a balloon containing carbon monoxide. The reaction was heated under refluxed for 16 h and then brine (10 mL) was added and the mixture extracted into ethyl acetate (2 × 30 mL). The organic layer was dried (Na_2SO_4), filtered and evaporated and the product was purified over silica gel eluting with diethyl ether/petrol (1:1) to afford **9** (13 mg 15%) as a colourless solid (mp. 171-172 ^oC) and **10** (36 mg 42%) as a colourless solid (mp. 188-189 ^oC).

Ethyl 3-(all *cis*-2,3,5,6-tetrafluorocyclohexyl)benzoate (9)

^{1}H NMR (300 MHz, $CDCl_3$) δ_H 8.06 - 8.02 (2H, m, C**H**-2, C**H**-6), 7.81 (1H, dt, *J* 7.6, 1.4 Hz C**H**-4), 7.48 (1H, td, *J* 7.8, 0.4 Hz C**H**-5), 5.14 - 4.90 (2H, m, C**H**F-2\`), 4.80 - 4.50 (2H, m, C**H**F-3\`), 4.39 (2H, q, *J* 7.1 Hz, C**H**$_2$CH$_3$) 2.81 - 2.48 (3H, m, C**H**-1, C**H**$_A$**H**$_B$-4), 1.40 (3H, t *J* 7.2 Hz, CH$_2$C**H**$_3$),; **^{13}C NMR** (125 MHz, $CDCl_3$) δ_C 166.2 (s, **C**=O), 135.8 (s, **C**H-1), 133.3 (t, *J* 3.6 Hz **C**H-3), 130.9 (s, 1C, **C**H-2), 130.2 (s, 1C, **C**H-4), 129.3 (s, 1C, **C**H-6), 129.1 (s, 1C, **C**H-5), 89.7 – 88.0 (m, **C**HF-2\`), 88.1 – 86.2 (m, **C**HF-3\`), 61.1 (s, **C**H$_2$CH$_3$), 44.1 – 43.7 (m, **C**H-1\`), 27.1 (tt, *J* 22.0, 2.6 Hz, **C**H$_2$-4\`), 14.3 (s, CH$_2$**C**H$_3$); **^{19}F{^{1}H} NMR** (282 MHz, $CDCl_3$) δ_F –190.8 (2F, dd, *J* 7.8, 5.1 Hz, CH**F**-2\`), –210.3 (2F, dd, *J* 7.8, 5.0 Hz, CH**F**-3\`); HRMS (**ESI$^+$**) *m/z* $[M+Na]^+$ calcd for $C_{15}H_{16}F_4NaO_2^+$: 327.0984, found 327.0975.

Ethyl 4-(all *cis*-2,3,5,6-tetrafluorocyclohexyl)benzoate (10)

^{1}H NMR (300 MHz, $CDCl_3$) δ_H 8.05 (2H, d, *J* 8.4 Hz, C**H**-3,), 7.56 (2H, d, *J* 8.2 Hz, C**H**-2), 5.13 - 4.90 (2H, m, C**H**F-2\`), 4.80 - 4.66 (2H, m, C**H**F-3\`), 4.39 (2H, q, *J* 7.2 Hz, C**H**$_2$CH$_3$) 2.86 - 2.43 (3H, m, C**H**-1, C**H**$_A$**H**$_B$-4), 1.39 (3H, t, *J* 7.2 Hz, CH$_2$C**H**$_3$); **^{13}C NMR** (125 MHz, $CDCl_3$) δ_C 166.2 (s, **C**=O), 140.3 (s, **C**H-1), 130.3 (s, **C**H-4), 130.1 (s, 2C, **C**H-2), 129.2 (t, 2C, *J* 2.7Hz, **C**H-3), 89.5 - 87.5 (m, **C**HF-2\`), 88.0 - 86.05 (m, **C**HF-3\`), 61.1 (s, **C**H$_2$CH$_3$), 44.2 - 43.7 (m, **C**H-1\`), 27.1 (tt, *J* 22.3, 2.7 Hz, **C**H$_2$-4\`), 14.3 (s, CH$_2$**C**H$_3$); **^{19}F{^{1}H} NMR** (282 MHz, $CDCl_3$) δ_F -190.7 (2F, dd, *J* 8.1, 5.1 Hz, CH**F**-2\`), –210.1 (2F, dd, *J* 7.6, 5.1 Hz, CH**F**-3\`); HRMS (**ESI$^+$**) *m/z* $[M+Na]^+$ calcd for $C_{15}H_{16}F_4NaO_2^+$: 327.0984, found 327.0975.

Ethyl 2-(all *cis*-2,3,5,6-tetrafluorocyclohex-1-yl)benzoate (8)

The same procedure used for as **9** and **10** was carried our using **5** (100 mg, 0.279 mmol). This generated **8** (74 mg, 88%) as a colourless solid, mp. 160–161 ^oC.

^{1}**H NMR** (300 MHz, $CDCl_3$) δ_H 8.07 - 8.03 (2H, m, , C**H**-3, C**H**-6), 7.58 (1H, dt, *J* 1.43, 7.6 Hz, C**H**-5), 7.42 (1H, dt, *J* 1.2, 7.8 Hz, C**H**-4), 5.14 - 4.92 (2H, m, C**H**F-2`), 4.85 - 4.55 (2H, m, C**H**F-3`), 4.33 (2H, q, *J* 7.2 Hz, CH_2CH$_3$), 4.06 (1H, tt, *J* 1.1, 38.0 Hz, C**H**-1`), 2.85 - 2.67 (1H, m, C$H_A$H$_B$-4`), 2.54 - 2.44 (1H, m, CH$_A$H$_B$-4`), 1.39 (3H, t *J* 7.2 Hz, CH$_2$CH$_3$); $^{13}$**C NMR** (125.7 MHz, $CDCl_3$) $\delta_C$ 167.6 (s, **C**=O), 138.1 (s, **C**H-1), 132.9 (s, 2C, **C**H-3), 131.3 (t, *J* 6.5 Hz **C**H-2), 131.1 (s, 1C, **C**H-6), 128.3 (s, 1C, **C**H-5), 127.4 (s, 1C, **C**H-4), 90.4 - 88.8 (m, **C**HF-2`), 88.0 – 86.2 (m, **C**HF-3`), 61.3 (s, **C**H$_2$CH$_3$), 38.4-38.1 (m, **C**H-1`), 27.4 (tt, *J* 22.2, 2.6 Hz, **C**H$_2$-4`), 14.2 (s, CH$_2$**C**H$_3$); $^{19}$**F{$^1$H} NMR** (282 MHz, $CDCl_3$) $\delta_F$ –189.5 (2F, dd, *J* 7.8, 5.2 Hz, CH**F**-2`), –209.7 (2F, dd, *J* 7.9, 5.2 Hz, CH**F**-3`); HRMS (**ESI$^+$**) *m/z* [M+Na]$^+$ calcd for $C_{15}H_{16}F_4NaO_2^+$: 327.0984, found 327.0973.

2-(All *cis*-2,3,5,6-tetrafluorocyclohex-1-yl)benzoic acid (11)

A solution of ethyl benzoate **8** (60 mg, 0.148 mmol) in a mixture of TFA and water, (9:1, 3 mL), was heated at 100 ºC for 24 h. The reaction was cooled to ambient temperature and then diluted with water (10 mL). The mixture was washed with ethyl acetate (2 × 20 mL) and the organics were collected, dried ($MgSO_4$) and evaporated. The product was purified over silica gel eluting with ethyl acetate/petrol (2:1) containing acetic acid (1%). This afforded benzoic acid **11** (51 mg, 94%) as colourless solid. Mp. 244–245 ºC.

^{1}**H NMR** (500 MHz, CD_3COCD_3) δ_H 8.09 (1H, dd, *J* 1.5, 8.0 Hz, C**H**-6), 8.04 (1H, d, *J* 8.0 Hz, C**H**-3), 7.65 (1H, td, *J* 1.37, 7.6 Hz, C**H**-5), 7.50 (1H, td, *J* 1.0, 7.6 Hz, C**H**-4), 5.20 - 5.07 (2H, m, C**H**F-2`), 5.06 - 4.90 (2H, m, C**H**F-3`), 4.17 (1H, tt, *J* 1.5, 38.8 Hz, C**H**-1`), 2.63 - 2.55 (1H, m, C$H_A$H$_B$-4`), 2.54 - 2.48 (1H, m, CH$_A$H$_B$-4`); $^{13}$**C NMR** (125.7 MHz, $CDCl_3$) $\delta_C$ 168.2 (s, **C**=O), 138.8 (s, **C**H-1), 132.3 (s, 2C, **C**H-3), 131.1 (t, *J* 6.4 Hz **C**H-2), 131.0 (s, 1C, **C**H-6), 129.3 (s, 1C, **C**H-5), 127.6 (s, 1C, **C**H-4) 91.0 - 89.2 (m, **C**HF-2`), 87.8 - 86.4 (m, **C**HF-3`), 38.5 (m, **C**H-1`), 27.3 (tt, *J* 22.0, 2.8 Hz, **C**H$_2$-4); ^{19}**F{^{1}H} NMR** (470 MHz, CD_3COCD_3) δ_F –190.5 (2F, dd, *J* 7.6, 5.2 Hz, CH**F**-2`), –210.9 (2F, dd, *J* 7.2, 5.1 Hz, CH**F**-3`); HRMS (**ESI$^-$**) *m/z* [M-H]$^-$ calcd for $C_{13}H_{11}F_4O_2^-$: 275.0773, found 275.0701.

3-(All *cis*-2,3,5,6-tetrafluorocyclohexyl)benzoic acid (12)

Following the procedure described for the preparation of **11**, ester **9** (60 mg, 0.148 mmol) afforded carboxylic acid **12** (48 mg, 89%) as colourless solid, mp. 271–272 ºC.

^{1}**H NMR** (400 MHz, CD_3COCD_3) δ_H 8.26 (1H, bs, C**H**-2), 8.03 (1H, dt, *J* 7.7, 1.3 Hz C**H**-6), 7.85 (1H, d, *J* 7.7 Hz, C**H**-4), 7.48 (1H, t, *J* 7.6 Hz, C**H**-5), 5.29 - 5.10 (2H, m, C**H**F-2`), 5.06 - 4.91 (2H, m, C**H**F-3`), 3.33 (1H, t, *J* 38.5 Hz, C**H**-1), 2.63 - 2.47 (2H, m, , CH_AH_B-4); ^{13}**C NMR** (100.6 MHz, CD_3COCD_3) δ_C 166.5 (s, **C**=O), 137.6 (s, **C**H-1), 133.8 (t, *J* 2.7 Hz **C**H-3), 130.8 (s, 1C, **C**H-2), 130.6 (s, 1C, **C**H-4), 128.7 (s, 1C, **C**H-6), 128.6 (s, 1C, **C**H-5), 90.8 -

88.6 (m, **C**HF-2`), 88.2 - 86.0 (m, **C**HF-3`), 43.0 - 42.6 (m, **C**H-1`), 27.0 (tt, *J* 22.0, 2.8 Hz, **C**$H_2$-4`); **^{19}F{^{1}H} NMR** (376.6 MHz, CD_3COCD_3) δ_F –191.4 (2F, dd, *J* 7.0 4.8 Hz, CH**F**-2`), –211.0 (2F, dd, *J* 8.0, 5.6 Hz, CH**F**-3`); HRMS (**ESI$^-$**) *m/z* [M-H]$^-$ calcd for $C_{13}H_{11}F_4O_2^-$: 275.0773, found 275.0701.

4-(All *cis*-2,3,5,6-tetrafluorocyclohexyl)benzoic acid (13)

Following the same procedure for the preparation of **11**, ester **10** (60 mg, 0.148 mmol) afforded **13** (49 mg, 90%) as colourless solid, mp. 269–270 °C. **^{1}H NMR** (400 MHz, CD_3COCD_3) δ_H 8.06 (2H, d, *J* 8.6 Hz, C**H**-3,), 7.71 (2H, d, *J* 8.0, C**H**-2), 5.30 - 5.12 (2H, m, C**H**F-2`), 5.10 - 4.90 (2H, m, C**H**F-3`), 3.34 (1H, tt, *J* 38.2, 1.8 Hz, C**H**-1), 2.63 - 2.46 (2H, m, CH_A**H**$_B$-4); **^{13}C NMR** (125 MHz, CD_3COCD_3) δ_C 166.5 (s, **C**=O), 142.1 (s, **C**H-1), 129.6 (s, **C**H-4), 129.6 (s, 2C, **C**H-2), 129.4 (t, 2C, *J* 2.7 Hz, **C**H-3), 90.4 - 88.6 (m, **C**HF-2`), 87.9 - 86.2 (m, **C**HF-3`), 43.1 - 42.7 (m, **C**H-1`), 27.0 (tt, *J* 22.0, 2.8 Hz, **C**$H_2$-4`); **^{19}F{^{1}H} NMR** (370 MHz, CD_3COCD_3) δ_F –191.43 (2F, dd, *J* 7.1, 4.6 Hz, CH**F**-2`), –210.8 (2F, dd, *J* 8.2, 5.6 Hz, CH**F**-3`); HRMS (**ESI$^-$**) *m/z* [M-H]$^-$ calcd for $C_{13}H_{11}F_4O_2^-$: 275.0773, found 275.0701.

3-(All *cis*-2,3,5,6-tetrafluorocyclohex-1-yl)benzaldehyde (14), 4-(all *cis*-2,3,5,6-tetrafluorocyclohex-1-yl)benzaldehyde (15)

A flame-dried flask was charged with a solution of aryl iodides **6**/**7** (1.3 g, 3.63 mmol) and $Pd(PPh_3)_4$ (210 mg, 4.0 mol %) in THF (4 mL). The flask was evacuated and fixed with a balloon containing carbon monoxide and the reaction was heated at 50 °C. A solution of tributyl tin hydride (1.16 g, 4.0 mmol) in dry THF (10 mL) was added dropwise by syringe over 3 h. Upon completion of the addition, the reaction was dilute by $NaHCO_3$ (20 mL) and the product extracted into diethyl ether. The organics were dried (Na_2SO_4), filtered and the solvent removed. Purification over silica gel, eluting with petrol/ethyl acetate/diethyl ether, (7:2.5:0.5 respectively), afforded benzaldehyde **14** (167 mg, 17%) and **15** (492 mg, 52%) as colorless solids.

3-(All *cis*-2,3,5,6-tetrafluorocyclohex-1-yl)benzaldehyde (14).

Mp. 182-183 °C, **^{1}H NMR** (400 MHz, $CDCl_3$) δ_H 10.02 (1H, s, C**H**O), 7.97 (1H, bs, C**H**-2), 7.87 (1H, dt, *J* 7.7, 1.4 Hz, C**H**-6), 7.81 (1H, d, *J* 7.8 Hz, C**H**-4), 7.57 (1H, t, *J* 7.6 Hz, C**H**-5), 5.14 - 4.92 (2H, m, C**H**F-2`), 4.82 - 4.54 (2H, m, C**H**F-3`), 2.88 - 2.62 (2H, m, C**H**-1, C**H**$_A$$H_B$-4), 2.56 – 2.45 (1H, m, CH_A**H**$_B$-4); **^{13}C NMR** (125.7 MHz, $CDCl_3$) δ_C 192.1 (s, **C**=O), 136.8 (s, **C**H-1), 136.7 (t, *J* 2.6 Hz, **C**H-3), 135.4 (s, 1C, **C**H-2), 130.3 (s, 1C, **C**H-4), 129.7 (s, 1C, **C**H-6), 129.5 (s, 1C, **C**H-5), 89.7 - 87.9 (m, **C**HF-2`), 87.7 - 85.9 (m, **C**HF-3`), 43.9 - 43.6 (m, **C**H-1`), 27.0 (tt, *J* 22.0, 2.6 Hz, **C**$H_2$-4`); **^{19}F{^{1}H} NMR** (282.3 MHz, $CDCl_3$) δ_F –190.4 (2F, dd,

J 7.8, 5.2 Hz, CH**F**-2\`), –209.7 (2F, dd, *J* 7.8, 5.2 Hz, CH**F**-3\`); HRMS (**ESI$^+$**) *m/z* $[M+MeOH+Na]^+$ calcd for $C_{14}H_{16}F_4NaO_2^+$: 315.0984, found 315.0975.

4-(All *cis*-2,3,5,6-tetrafluorocyclohex-1-yl)benzaldehyde (15)

Mp. 218-220 ºC. **^{1}H NMR** (400 MHz, $CDCl_3$) δ_H 10.04 (1H, s, C**H**O), 7.91 (2H, d, *J* 8.4 Hz, C**H**-3), 7.68 (2H, d, *J* 8.0 Hz, C**H**-2), 5.11 - 4.95 (2H, m, C**H**F-2\`), 4.77 - 4.54 (2H, m, C**H**F-3\`), 2.85 - 2.46 (3H, m, C**H**-1, C$H_A$$H_B$-4); **^{13}C NMR** (100.6 MHz, $CDCl_3$) δ_C 191.7 (s, **C**=O), 142.0 (s, **C**H-1), 136.1 (s, **C**H-4), 130.1 (s, 2C, **C**H-2), 130.0 (t, 2C, *J* 2.3 Hz, **C**H-3), 89.5 - 87.4 (m, **C**HF-2\`), 87.7 - 85.7 (m, **C**HF-3\`), 44.4 - 44.1 (m, **C**H-1\`), 27.1 (tt, *J* 22.1, 2.7 Hz, **C**$H_2$-4\`); **^{19}F{^{1}H} NMR** (376.6 MHz, $CDCl_3$) δ_F –190.2 (2F, dd, *J* 7.4, 5.1 Hz, CH**F**-2\`), –209.5 (2F, dd, *J* 7.8, 5.4 Hz, CH**F**-3\`); HRMS (**ESI$^+$**) *m/z* $[M+MeOH+Na]^+$ calcd for $C_{14}H_{16}F_4NaO_2^+$: 315.0984, found 315.0974.

1,2-Bis(4-(all-*cis*-2,3,5,6-tetrafluorocyclohexyl)phenyl)ethane (17)

A solution of $TiCl_4$ in DCM (1.0 M) (0.45 mL, 0.45 mmol) was added dropwise to a suspension of Zn (30 mg, 0.46 mmol) in anhydrous THF (2 mL) at 0 ºC. The resulting mixture was heated at reflux for 1 h. After cooling to 0 ºC, a solution of aldehyde **15** (40 mg, 0.15 mmol) in anhydrous THF (2 mL) was added, and the reaction was heated at reflux for 16 h. The reaction was then poured into a mixture of a saturated $NaHCO_3$ (5 mL) solution and DCM (5 mL) and was stirred for 3 h. After filtration through a Celite pad, and washing with hot chloroform, the layers were separated. The aqueous layer was extracted into ethyl acetate and the combined extracts were dried (Na_2SO_4), filtered and the solvent removed. The product was taken up in ethyl acetate (10 mL) and was directly hydrogenated by addition of Pd/C catalyst (5 mg) and stirred under an atmosphere of hydrogen. The reaction was stirred for 16h at 20 °C and was then filtered through a Celite pad and the solvent evaporated. Purification over silica gel eluting with petrol/ethyl acetate (1:1) afford dihydrostilbene **17** (29 mg, 76% over 2 steps) as a colourless solid.

Mp. 158-159 ºC.**^{1}H NMR** (500 MHz, CD_3COCD_3) δ_H 7.48 (4H, d, *J* 8.0 Hz, C**H**-3), 7.30 (4H, d, *J* 8.2, C**H**-2), 5.19 - 5.06 (4H, m, C**H**F-2\`), 5.05 - 4.89 (4H, m, C**H**F-3\`), 3.16 (2H, t, *J* 38.8 Hz, C**H**-1), 2.96 (4H, s, 2PhC**H$_2$**), 2.61 - 2.46 (4H, m, C**H$_A$H$_B$**-4); **^{13}C NMR** (125.6 MHz, CD_3COCD_3) δ_C, 141.1 (s, 2C, **C**H-1), 134.6 (s, 2C, **C**H-4), 129.2 (s, 4C, **C**H-2), 128.4 (s, 4C, *J* 2.3 Hz, **C**H-3), 90.8 - 89.1 (m, **C**HF-2\`), 88.1 - 86.3 (m, **C**HF-3\`), 42.8 - 42.5 (m, **C**H-1\`), 37.1 (s, 2C, **C**$H_2$), 27.1 (tt, *J* 21.8, 2.7 Hz, **C**$H_2$-4\`); **^{19}F{^{1}H} NMR** (470.3 MHz, CD_3COCD_3) δ_F –191.3 (4F, d, *J* 7.5, Hz, CH**F**-2\`), –210.8 (4F, dd, *J* 7.7, 5.6 Hz, CH**F**-3\`); HRMS (**ESI$^+$**) *m/z* $[M+K]^+$ calcd for $C_{26}H_{26}F_8K^+$: 529.1599, found 529.1743.

1-(All-*cis*-2,3,5,6-tetrafluorocyclohexyl)-4-vinylbenzene (18)

A solution of $TiCl_4$ in DCM (1.0 M) (2.2 mL, 2.2 mmol) was added to a suspension of zinc dust (88 mg, 1.4 mmol), and diiodomethane (121 mg, 0.45 mmol) in dry THF (2 mL) at 0 ºC. An instantaneous reaction occurred which evolved heat and underwent a colour change to dark brown. After 15 min, a solution of aldehyde **15** (40 mg, 0.15 mmol) in THF (2 mL) was added dropwise and the resulting mixture was stirred at 25 ºC for 12 h. The reaction was diluted with diethyl ether, poured into 1 M hydrochloric acid (10 mL) and was then extracted into diethyl ether. The separated organic layers were washed with brine (2 × 20 mL), dried (Na_2SO_4), and the solvent removed. The product was purified over silica gel eluting with petrol/ethyl acetate (2:1) to give styrene **18** as colourless solid (31 mg, 79%).

Mp. 154-155 ºC . **^{1}H NMR** (500 MHz, $CDCl_3$) δ_H 7.44 (2H, d, *J* 8.4 Hz, C**H**-2,), 7.42 (2H, d, *J* 8.0, C**H**-3), 6.72 (1H, dd, *J* 17.6 10.8 Hz, C**H**=CH_AH_B), 5.77 (1H, d, *J* 17.5 Hz, C**H**=C$\mathbf{H_A}H_B$), 5.28 (1H, d, *J* 10.8 Hz, C**H**=$CH_A\mathbf{H}_B$), 5.07 - 4.92 (2H, m, C**H**F-2), 4.73 - 4.53 (2H, m, C**H**F-3), 2.86 - 2.72 (1H, m, C$\mathbf{H_A}H_B$-1), 2.60 (1H, t, *J* 37.2 Hz, C**H**-4), 2.50 – 2.45 (1H, m, $CH_A\mathbf{H_B}$-1); **^{13}C NMR** (125.7 MHz, $CDCl_3$) δ_C 137.4 (s, **C**H-1), 136.1 (s, **C**H=CH_2), 135.0 (m, **C**H-4), 129.4 (s, 2C, **C**H-2), 126.6 (s, 2C, **C**H-3), 114.5 ((s, CH=**C**H_2), 89.8 - 88.1 (m, **C**HF-2\`), 87.7 - 86.2 (m, **C**HF-3\`), 43.9 (m, **C**H-1\`), 27.1 (tt, *J* 22.0, 2.9 Hz, **C**$H_2$-4\`); **^{19}F{^{1}H} NMR** (470.4 MHz, $CDCl_3$) δ_F –190.2 (2F, dd, *J* 8.0, 5.8 Hz, CH**F**-2), –209.7 (2F, dd, *J* 7.7, 5.8 Hz, CH**F**-3); HRMS (**ESI$^+$**) *m/z* $[2M+CH_3OH+5H]^+$ calcd for $C_{14}H_{14}F_4^+$: 553.2064, found 553.3893.

(4-(All-*cis*-2,3,5,6-tetrafluorocyclohexyl)phenyl)methanol (19)

$NaBH_4$ (88 mg, 2.3 mmol) was added to a solution of benzaldehyde **15** (400 mg, 1.53 mmol) in dry THF (10 mL). The reaction was stirred at ambient temperature for 1 h, and was quenched by the addition of solid NH_4Cl (150 mg) followed by water (10 mL). The product was extracted into ethyl acetate (2 × 30 mL), washed with brine and dried (Na_2SO_4). The solvent was removed under reduced pressure and the product purified over silica gel, eluting with petrol/ethyl acetate (1:1) to give benzyl alcohol **19** as a colourless solid (398 mg, 98%).

Mp. 184-185 ºC .**^{1}H NMR** (500 MHz, CD_3COCD_3) δ_H 7.52 (2H, d, *J* 8.0 Hz, C**H**-3,), 7.38 (2H, d, *J* 8.2 Hz, C**H**-2), 5.22 - 5.05 (2H, m, C**H**F-2\`), 5.05 - 4.89 (2H, m, C**H**F-3\`), 4.65 (2H, d, *J* 6.0 Hz, PhC$\mathbf{H_2}$), 4.21 (1H, t, *J* 5.8 Hz, O**H**), 3.18 (1H, t, *J* 38.8 Hz, C**H**-1), 2.62 - 2.43 (2H, m, C$\mathbf{H_A}\mathbf{H_B}$-4); **^{13}C NMR** (125.6 MHz, CD_3COCD_3) δ_C 141.8 (s, **C**H-1), 135.5 (s, **C**H-4), 129.0 (s, 2C, **C**H-2), 126.6 (s, 1C, **C**H-3), 90.8 - 89.0 (m, **C**HF-2\`), 88.1 - 86.3 (m, **C**HF-3\`), 63.4 (s, 1C, Ph**C**H_2), 42.9 - 42.5 (m, **C**H-1\`), 27.1 (tt, *J* 22.1, 3.0 Hz, **C**$H_2$-4\`); **^{19}F{^{1}H} NMR** (470 MHz, CD_3COCD_3) δ_F –191.3 (2F, dd, *J* 7.5, 5.6 Hz, CH**F**-2\`), –210.8 (2F, dd, *J* 7.6, 5.6 Hz, CH**F**-3\`); HRMS (**ESI$^+$**) *m/z* $[M+Na]^+$ calcd for $C_{13}H_{14}F_4NaO^+$: 285.0878, found 285.0870.

(3-(All-*cis*-2,3,5,6-tetrafluorocyclohexyl)phenyl)methanol (28)

Following the same procedure for the reduction of **15,** treatment of benzaldehyde **14** (260 mg, 1.0 mmol) afforded benzyl alcohol **28** as colourless solid (258 mg, 98%) mp. 159–160 ºC. **^{1}H NMR** (500 MHz, $CDCl_3$) δ_H 7.48 (1H, bs, C**H**-2), 7.44-7.35 (3H, m, C**H**-4, C**H**-5, C**H**-6), 5.07 - 4.96 (2H, m, C**H**F-2\`), 4.72 (2H, s, PhC**H**$_2$), 4.70 - 4.58 (2H, m, C**H**F-3\`), 2.81 - 2.72 (1H, m, C**H**$_A$H$_B$-4), 2.62 (1H, t, *J* 37.0 Hz, C**H**-1), 2.51 - 2.44 (1H, m, CH$_A$**H**$_B$-4), 1.63 (1H, bs, PhCH$_2$O**H**); **^{13}C NMR** (125.7 MHz, $CDCl_3$) δ_C 141.5 (s, **C**H-1), 135.9 (s, **C**H-3), 129.1 (s, 1C, **C**H-2), 128.5 (s, 1C, **C**H-4), 127.7 (s, 1C, **C**H-6), 126.7 (s, 1C, **C**H-5), 89.9 - 88.1 (m, **C**HF-2\`), 87.9 - 86.1 (m, **C**HF-3\`), 65.1(s, Ph**C**H$_2$), 44.1 - 43.8 (m, **C**H-1\`), 27.1 (tt, *J* 22.2, 2.8 Hz, **C**H$_2$-4\`); **^{19}F{^{1}H} NMR** (470.3 MHz, $CDCl_3$) δ_F –190.2 (2F, dd, *J* 7.5, 4.5 Hz, CH**F**-2\`), –209.6 (2F, dd, *J* 7.7, 4.3 Hz, CH**F**-3\`); HRMS (**ESI$^+$**) *m/z* $[M+Na]^+$ calcd for $C_{13}H_{14}F_4NaO^+$: 285.0878, found 285.0871.

1-(Chloromethyl)-4-(all-*cis* -2,3,5,6-tetrafluorocyclohexyl)benzene (23)

Mesylchloride (0.007 mL, 0.091 mmol) was added dropwise to a stirred solution of p-benzyl alcohol **19** (20 mg, 0.076 mmol) in dry DCM (2 mL) and trimethylamine (0.02 mL, 0.152 mmol) at −78 ºC. The reaction was stirred for 16 h at −78 ºC and was then warmed to ambient temperature, when DCM (10 mL) was added. The organics layers were washed with dilute HCl (5 mL) and then saturated $NaHCO_3$ (10 mL) and then dried (Na_2SO_4). After filtration through a Celite pad the organic solvent was evaporated under reduced pressure to afford **23** as colourless solid (18 mg, 85%).

Mp. 171-172 ºC; **^{1}H NMR** (300 MHz, $CDCl_3$) δ_H 7.48 (2H, d, *J* 8.1 Hz, C**H**-2), 7.40 (2H, d, *J* 8.2, C**H**-3), 5.11 - 4.88 (2H, m, C**H**F-2\`), 4.79 - 4.60 (4H, m, PhC**H**$_2$, C**H**F-3\`), 2.85 - 2.42 (3H, m, C**H**-1,C**H**$_A$**H**$_B$-4); **^{13}C NMR** (75 MHz, $CDCl_3$) δ_C 137.4 (s, **C**H-1), 135.8 (s, **C**H-4), 129.6 (s, 2C, **C**H-2), 129.0 (s, 1C, **C**H-3), 90.2 - 88.0 (m, 2C, **C**HF-2\`), 87.7 - 85.5 (m, 2C, **C**HF-3\`), 45.6 (s, 1C, Ph**C**H$_2$), 44.0 - 43.4 (m, 1C, **C**H-1\`), 27.1 (tt, 1C, *J* 22.2, 2.7 Hz, **C**H$_2$-4\`); **^{19}F{^{1}H} NMR** (282 MHz, $CDCl_3$) δ_F –190.2 (2F, dd, *J* 8.7, 5.2 Hz, CH**F**-2\`), -209.7 (2F, dd, *J* 7.6, 5.2 Hz, CH**F**-3\`); HRMS (**ESI$^+$**) *m/z* $[M+H-HCl]^+$ calcd for $C_{14}H_{13}ClF_4^+$: 245.0953, found 245.0943.

1-(Iodomethyl)-4-(all-*cis*-2,3,5,6-tetrafluorocyclohexyl)benzene (20)

An aqueous solution of HI (57%) (0.58 mL) was added dropwise to a solution of *p*-benzyl alcohol **19** (120 mg, 0.45 mmol) in chloroform (5 mL). The mixture was allowed to stir at room temperature for 30 h, and then the excess of iodine was destroyed by adding Na_2SO_3 solution. The product was extracted into chloroform (2 × 30 mL), and the organics were combined and washed with saturated $NaHCO_3$ solution and then dried (Na_2SO_4). Filtration and then removal of the organic solvent under reduced pressure gave benzyl iodide **20** as

colourless solid. This product was used directly without further purification (163 mg, 95%). Mp. 188–189 ºC. **^{1}H NMR** (300 MHz, $CDCl_3$) δ_H 7.40 (4H, bs, C**H**-3, C**H**-2), 5.09 - 4.87 (2H, m, C**H**F-2`), 4.78 - 4.47 (2H, m, C**H**F-3`), 4.45 (2H, s, PhC**H**$_2$), 2.83 - 2.42 (3H, m, C**H**-1,C**H**$_A$**H**$_B$-4); **^{13}C NMR** (125.75 MHz, $CDCl_3$) δ_C 139.2 (s, **C**H-1), 135.2 (s, **C**H-4), 129.7 (s, 2C, **C**H-2), 129.1 (s, 1C, **C**H-3), 89.7 - 87.7 (m, 2C, **C**HF-2`), 88.2 - 86.2 (m, 2C, **C**HF-3`), 43.9 - 43.5 (m, 1C, **C**H-1`), 27.1 (tt, 1C, *J* 22.1, 2.7 Hz, **C**H$_2$-4`), 4.8 (s, 1C, Ph**C**H$_2$); **^{19}F{^{1}H} NMR** (282 MHz, $CDCl_3$) δ_F –190.2 (2F, dd, *J* 7.7, 5.1 Hz, CH**F**-2`), –209.7 (2F, dd, *J* 7.8, 5.1 Hz, CH**F**-3`); HRMS (**ESI$^+$**) *m/z* $[M+H-HI]^+$ calcd for $C_{13}H_{14}IF_4^+$: 245.0953 found.

1-(Iodomethyl)-3-(all-*cis*-2,3,5,6-tetrafluorocyclohexyl)benzene (29)

Iodination of **28** (60 mg, 0.23 mmol) followed the procedure used for **19** to afford benzyl iodide **29** (79 mg, 94%) as colourless white solid.

Mp. 172-173 ºC; **^{1}H NMR** (500 MHz, $CDCl_3$) δ_H 7.48 (1H, bs, C**H**-2), 7.38 (2H, d, *J* 7.5 Hz, C**H**-4, C**H**-6), 7.32 (1H, d, *J* 7.5 Hz, C**H**-5), 5.05 - 4.95 (2H, m, C**H**F-2`), 4.72 - 4.54 (2H, m, C**H**F-3`), 4.46 (2H, s, PhC**H**$_2$), 2.80 - 2.71 (1H, m, C**H**$_A$H$_B$-4), 2.60 (1H, t, *J* 38.1 Hz C**H**-1), 2.51 - 2.45 (1H, m, , CH$_A$**H**$_B$-4); **^{13}C NMR** (125.7 MHz, $CDCl_3$) δ_C 139.9 (s, **C**H-1), 136.2 (s, **C**H-3), 129.5 (s, 1C, **C**H-2), 129.3 (s, 1C, **C**H-4), 128.8 (s, 1C, **C**H-6), 128.5 (s, 1C, **C**H-5), 89.7 - 88.0 (m, **C**HF-2`), 87.8 - 86.1 (m, **C**HF-3`), 43.9 - 43.6 (m, **C**H-1`), 27.1 (tt, *J* 22.2, 2.7 Hz, **C**H$_2$-4`), 5.1 (s, Ph**C**H$_2$); **^{19}F{^{1}H} NMR** (470.3 MHz, $CDCl_3$) δ_F –190.2 (2F, dd, *J* 7.5, 5.6 Hz, CH**F**-2`), –209.6 (2F, dd, *J* 7.7, 5.7 Hz, CH**F**-3`); HRMS (**ESI$^+$**) $[M+H-HI]^+$ calcd for $C_{13}H_{14}IF_4^+$: 245.0953 found.

1-(Azidomethyl)-4-(all-*cis*-2,3,5,6-tetrafluorocyclohexyl)benzene (21)

Tetrabutylammonium azide (80 mg, 0.28 mmol) was added to a solution of benzyl iodide **20** (70 mg, 0.188 mmol) in acetone/water (4:1, 3 mL). The mixture was stirred at 20 °C for 3 h, and was then diluted with water (10 mL). The product was extracted into ethyl acetate (3 × 10 mL) and the organics were combined and dried (Na_2SO_4) and the solvent removed under reduced pressure. The product was purified over silica gel, eluting with petrol/ethyl acetate (2:1), to afford benzyl azide **21** as colourless solid (51 mg, 94%).

Mp. 149-150 ºC; **^{1}H NMR** (300 MHz, $CDCl_3$) δ_H 7.51 (2H, d, *J* 8.1 Hz, C**H**-2), 7.34 (2H, d, *J* 8.2, C**H**-3), 5.11 - 4.89 (2H, m, C**H**F-2`), 4.78 - 4.49 (2H, m, C**H**F-3`), 4.35 (2H, bs, PhC**H**$_2$), 2.84 - 2.42 (3H, m, C**H**-1,C**H**$_A$**H**$_B$-4); **^{13}C NMR** (125.75 MHz, $CDCl_3$) δ_C 135.6 (s, **C**H-1), 135.4 (s, **C**H-4), 129.7 (s, 2C, **C**H-2), 128.6 (s, 1C, **C**H-3), 89.8 - 87.7 (m, 2C, **C**HF-2`), 88.2 - 86.1 (m, 2C, **C**HF-3`), 54.3 (s, 1C, Ph**C**H$_2$), 43.9 – 43.5 (m, 1C, **C**H-1`), 27.1 (tt, 1C, *J* 22.2, 2.7 Hz, **C**H$_2$-4`; **^{19}F{^{1}H} NMR** (282 MHz, $CDCl_3$) δ_F –190.2 (2F, dd, *J* 7.7, 5.2 Hz, CH**F**-2`), –209.7 (2F, dd, *J* 7.8, 5.2 Hz, CH**F**-3`); HRMS (**ESI$^+$**) *m/z* $[M+H-N_2]^+$ calcd for $C_{13}H_{14}F_4N_3^+$: 260.1141, found 260.1055.

1-(Azidomethyl)-3-(all-*cis*-2,3,5,6-tetrafluorocyclohexyl)benzene (30)

m-Benzyl iodide **29** (60 mg, 0.16 mmol) was treated following the procedure for the preparation **21** above to afford *m*-benzyl azide **28** as colourless solid (42 mg, 91%).

Mp. 145-146 ºC; **^{1}H NMR** (500 MHz, $CDCl_3$) δ_H 7.47 - 7.39 (3H, m, C**H**-2, C**H**-4, C**H**-6), 7.32 (1H, d, *J* 7.4 Hz, C**H**-5), 5.08 - 4.94 (2H, m, C**H**F-2`), 4.73 - 4.54 (2H, m, C**H**F-3`), 4.37 (2H, s, PhC**H**$_2$), 2.82 - 2.73 (1H, m, C**H**$_A$H$_B$-4), 2.63 (1H, t, *J* 36.8 Hz C**H**-1), 2.51 – 2.46 (1H, m, CH$_A$**H**$_B$-4); **^{13}C NMR** (125.7 MHz, $CDCl_3$) δ_C 136.2 (s, **C**H-1), 136.1 (s, **C**H-3), 129.4 (s, 1C, **C**H-2), 129.1 (s, 1C, **C**H-4), 129.0 (s, 1C, **C**H-6), 127.9 (s, 1C, **C**H-5), 89.8 - 88.1 (m, **C**HF-2`), 87.9 - 86.1 (m, **C**HF-3`), 54.6(s, Ph**C**H$_2$), 44.1 - 43.8 (m, **C**H-1`), 27.1 (tt, *J* 22.0, 2.6 Hz, **C**H$_2$-4`); **^{19}F{^{1}H} NMR** (470.3 MHz, $CDCl_3$) δ_F –190.2 (2F, dd, *J* 7.5, 4.5 Hz, CH**F**-2`), –209.6 (2F, dd, *J* 7.7, 4.3 Hz, CH**F**-3`); HRMS (**ESI$^+$**) *m/z* $[M+H-N_2]^+$ calcd for $C_{13}H_{14}F_4N_3{}^+$: 260.1141, found 260.1058.

(4-(All-*cis*-2,3,5,6-tetrafluorocyclohexyl)phenyl)methanamine hydrochloride (27)

A solution of Ph_3P (93 mg, 0.35 mmol) in a THF:H_2O (10:1, 3 mL) mixture, was added directly to azide **21** (50 mg, 0.174 mmol) and the reaction was followed by TLC. The solvent was then removed under reduced pressure and the residue dissolved in CH_2Cl_2 (10 mL) and then 1 M HCl (10 mL) was added. The reaction mixture was left to stir for 1 h, and was then washed with CH_2Cl_2, and the acidic aqueous layer was collected. The solvent was removed under reduced pressure to afford benzyl amine hydrochloride **25** (38 mg, 74%) as a colourless solid.

Mp. 296-298 ºC. **^{1}H NMR** (500 MHz, d$_6$-DMSO) δ_H 8.49 (3H, bs, NH_3Cl), 7.51 (4H, bs, C**H**-2, C**H**-3), 5.23 - 5.05 (2H, m, C**H**F-2`), 5.05 - 4.85 (2H, m, C**H**F-3`), 4.01 (2H, bd, *J* 3.5 Hz PhC**H**$_2$), 3.24 (1H, t, *J* 39.4 Hz, C**H**-1), 2.46 - 2.28 (2H, m, C**H**-1,C**H**$_A$**H**$_B$-4); **^{13}C NMR** (125.75 MHz, d$_6$-DMSO) δ_C 137.5 (s, **C**H-1), 133.5 (s, **C**H-4), 129.5 (s, 2C, **C**H-2), 129.5 (s, 1C, **C**H-3), 90.8 - 89.3 (m, 2C, **C**HF-2`), 88.0 - 86.6 (m, 2C, **C**HF-3`), 45.7 (s, 1C, Ph**C**H$_2$), 42.2 - 41.8 (m, 1C, **C**H-1`), 27.3 (t, 1C, *J* 21.7 Hz, **C**H$_2$-4`); **^{19}F{^{1}H} NMR** (282 MHz, d$_6$-DMSO) δ_F –192.4 (2F, dd, *J* 7.8, 5.3 Hz, CH**F**-2`), –211.5 (2F, dd, *J* 7.7, 5.2 Hz, CH**F**-3`); HRMS (**ESI$^+$**) *m/z* $[M+H-HCl]^+$ calcd for $C_{13}H_{17}ClF_4N^+$: 262.1141, found 262.1204.

Methyl (*S*)-2-((*tert*-butoxycarbonyl)amino)pent-4-ynoate (24)

Thionyl chloride (0.5 mL, 6.5 mmol) was added dropwise over 5 min to dry MeOH (5 mL) at 0 °C and the solution was stirred for 10 min before propargyl-L-glycine was added (200 mg, 1.77 mmol) in one portion. The reaction was stirred for 16 h at 20 °C. The solvent and the excess thionyl chloride were then removed under reduced pressure to give an oily residue. The residue was dissolved in MeCN (5 mL), and then Et_3N (0.271 mL, 1.9 mmol) and di-tert-

butyl pyrocarbonate (423 mg, 1.9 mmol) were added and the reaction was stirred for 2 h at ambient temperature. The solvent was then evaporated and the resulting residue suspended in 1 M $NaHSO_4$ (10 mL). The product was extracted into CH_2Cl_2 (3 × 20 mL) and the combined extracts were washed with saturated $NaHCO_3$ (5 mL) and dried (Na_2SO_4). The solution was filtered through a Celite pad and was evaporated under reduced pressure to afford the product which was purified over silica gel eluting with (ethyl acetate/petrol) (9:1) to give **27** as a colourless oil (308 mg, 81%).

^{1}H NMR (300 MHz, $CDCl_3$) δ_H 5.35 (2H, d, *J* 7.2 Hz, N**H**Boc), 4.51 – 4.45 (1H, m, C**H**NHBoc), 3.77 (3H, s, COOC**H**$_3$), 2.73 (2H, m,HCCC**H**$_2$), 2.04 (1H, s,**H**CCCH$_2$), 1.45 (9H, bs, $(CH_3)_3$); **^{13}C NMR** (100.6 MHz, $CDCl_3$) δ_C 171.1, 155.1, 71.6, 60.4, 52.6, 51.9, 28.3, 22.8, 14.2.

Methyl(*S*)-2-((*tert*-butoxycarbonyl)amino)-4-(1-(3-(all-*cis*-2,3,5,6-tetrafluorocyclohexyl) benzyl)-1*H*-1,2,3-triazol-4-yl)propanoate (25)

A suspension of $Cu(OAc)_2$ (20 mol %) and sodium ascorbate (40 mol %) in H_2O (2 mL) was added to a solution of azide **21** (55 mg, 0.19 mmol), and propargyl-L-glycine **24** (41.0 mg, 0.19 mmol) in *tert*-butanol (4 mL), and the reaction was stirred for 16 h at 20 °C. Water (10 mL) was added and the product was extracted into ethyl acetate (3 × 30 mL). The combined extracts were washed with aqueous $NaHCO_3$ followed by brine and then dried (Na_2SO_4). The solvent was removed and the product purified over silica gel eluting with ethyl acetate/petrol (5:1) to afford the protected amino acid **25** as a white solid (71 mg, 72%).

Mp 176 – 178 ºC. $[\alpha]_D^{20}$ - 56.0 (c= 1 × 10^{-3}, DMSO); **^{1}H NMR** (300 MHz, $CDCl_3$) δ_H 7.48 (2H, d, *J* 8.2 Hz, C**H**-2,), 7.30 (1H, bs, **H**-triazol), 7.23 (2H, d, *J* 8.1, C**H**-3), 5.49 (3H, s, PhC**H**$_2$, N**H**Boc), 5.07 - 4.85 (2H, m, C**H**F-2`), 4.78 - 4.47 (3H, m, C**H**F-3`, C**H**NHBoc), 3.67 (3H, s, COOC**H**$_3$), 3.21 (2H, d, *J* 5.2 Hz, triazol-C**H**$_2$), 2.82 - 2.37 (3H, m, C**H**-1,C**H**$_A$**H**$_B$-4), 1.40 (9H, bs, $(CH_3)_3$ of Boc); **^{13}C NMR** (100.6 MHz, $CDCl_3$) δ_C 172.0 (s,1C, **C**OOMe), 155.4 (s,1C, NH**C**OO), 143.5 (s,1C, **C**N=N of triazol),136.2 (s, **C**H-1), 134.7 (s, **C**H-4), 130.0 (s, 2C, **C**H-2), 128.35 (s, 1C, **C**H-3), 122.1 (s,1C, **C**H-triazol), 89.9 - 87.7 (m, 2C, **C**HF-2`), 88.0 - 85.8 (m, 2C, **C**HF-3`), 53.6 (s, 1C, Ph**C**H$_2$), 53.0 (s,1C, **C**HNHBoc), 52.4 (s,1C, COO**C**H$_3$), 43.4 - 43.5 (m, 1C, **C**H-1`), 30.9 (s, 1C, **C**$(CH_3)_3$), 28.4 (s, 1C, triazol-**C**H$_2$), 28.2 (s, 3C, C(**C**H$_3$)$_3$), 27.1 (tt, 1C, *J* 22.2, 2.2 Hz, **C**H$_2$-4`); **^{19}F{^{1}H} NMR** (282 MHz, $CDCl_3$) δ_F –190.3 (2F, dd, *J* 7.6, 5.0 Hz, CH**F**-2`), –209.7 (2F, dd, *J* 7.8, 5.2 Hz, CH**F**-3`); HRMS (**ESI$^+$**) *m/z* $[M+Na]^+$ calcd for $C_{24}H_{30}F_4N_4NaO_4^+$: 537.2101, found 537.2085.

Methyl (*S*)-2-((*tert*-butoxycarbonyl)amino)-3-(1-(3-(all-*cis*-2,3,5,6-tetrafluorocyclohexyl) benzyl)-1*H*-1,2,3-triazol-4-yl)propanoate (31)

Triazole **31** (58 mg, 81%) was prepared as colourless solid following the procedure used above for **25**, and using *m*- benzylazide **30** (40 mg, 0.14 mmol).

Mp.130 – 131 ºC. $[\alpha]_D^{20}$ -96.0 (c= 5 × 10^{-4}, DMSO); ^{1}**H NMR** (400 MHz, $CDCl_3$) δ_H 7.50 (1H, d, *J* 7.6 Hz, C**H**-2), 7.38 (1H, t, *J* 7.6, C**H**-5), 7.31 (1H, s, **H**-triazol), 7.20 (2H, d, *J* 7.7, C**H**-4, C**H**-6), 5.51 (3H, s, PhCH_2, N**H**Boc), 5.05 - 4.89 (2H, m, C**H**F-2\`), 4.73 - 4.50 (3H, m, C**H**F-3\`, C**H**NHBoc), 3.66 (3H, s, COOCH_3), 3.20 (2H, d, *J* 5.3 Hz, triazol-CH_2), 2.82 - 2.37 (3H, m, C**H**-1,CH_AH_B-4), 1.40 (9H, bs, $(CH_3)_3$ of Boc); ^{13}**C NMR** (125.7 MHz, $CDCl_3$) δ_C 172.0 (s,1C, **C**OOMe), 155.4 (s,1C, NH**C**OO), 143.5 (s,1C, **C**N=N of triazol), 136.8 (s, **C**H-1), 135.4 (s, **C**H-3), 129.6 (s, 2C, **C**H-2, **C**H-4), 128.4 (s, 1C, **C**H-6), 127.3 (s, 1C, **C**H-5), 122.1 (s,1C, **C**H-triazol), 89.7 - 88.1 (m, 2C, **C**HF-2\`), 87.6 - 86.1 (m, 2C, **C**HF-3\`), 53.8 (s, 1C, Ph**C**H_2), 53.0 (s,1C, **C**HNHBoc), 52.4 (s,1C, COO**C**H_3), 43.9 - 43.5 (m, 1C, **C**H-1\`), 29.7 (s, 1C, **C**$(CH_3)_3$), 28.4 (s, 1C, triazol-**C**$H_2$), 28.2 (s, 3C, C(**C**$H_3)_3$), 27.1 (tt, 1C, *J* 22.1, 2.4 Hz, **C**$H_2$-4\`); ^{19}**F{^{1}H} NMR** (376 MHz, $CDCl_3$) δ_F –190.3 (2F, t, *J* 8.4, Hz, CH**F**-2\`), –209.7 (2F, dd, *J* 9.0, 5.4 Hz, CH**F**-3\`); HRMS (**ESI$^+$**) *m/z* $[M+Na]^+$ calcd for $C_{24}H_{30}F_4N_4NaO_4^+$: 537.2101, found 537.2081.

(*S*)-2-Amino-3-(1-(4-(all-*cis*-2,3,5,6-tetrafluorocyclohexyl)benzyl)-1H-1,2,3-triazol-4-yl)propanoic acid hydrochloride (26)

A solution of **25** (60 mg, 0.116 mmol) in a mixture of HCl 6 M/1,4-dioxane (1:1, 6 mL) was stirred at 80 ºC for 48 h, until TLC (ethyl acetate:petrol; 2:1), indicted consumption of all starting material. The reaction was then diluted with water (10 mL) and the product extracted into ethyl acetate (2 × 15 mL). The organics layers were washed with water (20 mL) and the aqueous was collected and evaporated to afford **26** (49 mg, 96%) as a colourless hydrochloride salt.

Mp**.** Decomposed at 290-292 ºC. $[\alpha]_D^{20}$ - 44.0 (c= 1 × 10^{-3}, DMSO); ^{1}**H NMR** (500 MHz, d*6*-DMSO) δ_H 8.44 (3H, bs, NH_3Cl), 8.06 (1H, s, **H**-triazol), 7.48 (2H, d, *J* 8.0 Hz, C**H**-2,), 7.31 (2H, d, *J* 8.0, C**H**-3), 5.59 (2H, s, PhCH_2), 5.19 - 5.04 (2H, m, C**H**F-2\`), 5.03 - 4.85 (2H, m, C**H**F-3\`), 4.22 (1H, bs, C**H**NHBoc), 3.25-3.12 (3H, m, triazol-CH_2, C**H**-1), 2.46 - 2.29 (2H, m, CH_AH_B-4); ^{13}**C NMR** (125.7 MHz, d*6*-DMSO) δ_C 170.5 (s,1C, **C**OOH), 141.3 (s,1C, **C**N=N of triazol),137.2 (s, **C**H-1), 135.5 (s, **C**H-4), 129.6 (s, 2C, **C**H-2), 128.4 (s, 1C, **C**H-3), 124.5 (s,1C, **C**H-triazol), 90.8 - 89.1 (m, 2C, **C**HF-2\`), 88.1 - 86.6 (m, 2C, **C**HF-3\`), 52.8 (s, 1C, Ph**C**H_2), 52.0 (s,1C, **C**HNH_2), 42.9 - 41.7 (m, 1C, **C**H-1\`), 27.3 (tt, 1C, *J* 21.0, 3.2 Hz, **C**$H_2$-4\`), 26.5 (s, 1C, triazol-**C**H_2); ^{19}**F{^{1}H} NMR** (282 MHz, d*6*-DMSO) δ_F –189.4 (2F, dd, *J* 7.6, 5.6 Hz, CH**F**-2\`), –209.8 (2F, dd, *J* 7.5, 5.6 Hz, CH**F**-3\`); HRMS (**ESI$^+$**) *m/z* $[M+H-HCl]^+$ calcd for $C_{18}H_{22}F_4ClN_4O_2^+$: 401.1601, found 401.1589.

(*S*)-2-Amino-3-(1-(3-(all-*cis*-2,3,5,6-tetrafluorocyclohexyl)benzyl)-1*H*-1,2,3-triazol-4-yl)propanoic acid hydrochloride (32)

Hydrochloride salt **32** (32 mg, 94%) was prepared following the procedure for the preparation of **26** starting from **31** (40 mg, 0.07 mmol) to afford the free amino acid. Mp. Decomposed at 226 ºC. $[\alpha]_D^{20}$ - 78.0 (c= 5 × 10^{-4}, DMSO).

^{1}H NMR (500 MHz, d_6-DMSO) δ_H 8.51 - 8.46 (3H, bd, NH_3), 8.08 (1H, d, *J* 4.0 Hz, **H**-triazol), 7.30 (2H, m, C**H**-2, C**H**-6), 7.38 (1H, t, *J* 7.6, C**H**-5), 7.23 (1H, d, *J* 7.7, C**H**-4), 5.60 (2H, s, PhC$\mathbf{H}_2$), 5.19 - 5.05 (2H, m, C**H**F-2\`), 5.04 - 4.86 (2H, m, C**H**F-3\`), 4.22 - 4.18 (1H, m, C**H**NH_3), 3.32 (3H, m, C**H**-1, triazol-C$\mathbf{H}_2$), 2.45 - 2.30 (2H, m, C$\mathbf{H}_A\mathbf{H}_B$-4); **^{13}C NMR** (100.6 MHz, d_6-DMSO) δ_C 170.4 (s,1C, **C**OOH), 141.3 (s,1C, **C**N=N of triazol),137.9 (s, **C**H-1), 136.5 (s, **C**H-3), 129.3 (s, 2C, **C**H-2), 129.0 (s, 1C, **C**H-4), 128.9 (s, 1C, **C**H-5), 127.3 (s, 1C, **C**H-6), 124.5 (s,1C, **C**H-triazol), 90.9 - 88.9 (m, 2C, **C**HF-2\`), 88.4 - 86.4 (m, 2C, **C**HF-3\`), 53.2 (s, 1C, Ph**C**H_2), 52.0 (s,1C, **C**HNH_3), 42.4 - 41.9 (m, 1C, **C**H-1\`), 31.1 (s, 1C, triazol-**C**$H_2$), 27.3 (t, 1C, *J* 21.3 Hz, **C**$H_2$-4\`); **^{19}F{^{1}H} NMR** (470 MHz, d_6-DMSO) δ_F –189.5 (2F, dd, *J* 7.6, 5.6 Hz, CH**F**-2\`), –209.6 (2F, dd, *J* 7.6, 5.6 Hz, CH**F**-3\`); HRMS (**ESI$^+$**) *m/z* $[M+H-HCl]^+$ calcd for $C_{18}H_{22}F_4ClN_4O_2^+$: 401.1601, found 401.1580.

^{1}H NMR of 8 ($CDCl_3$)

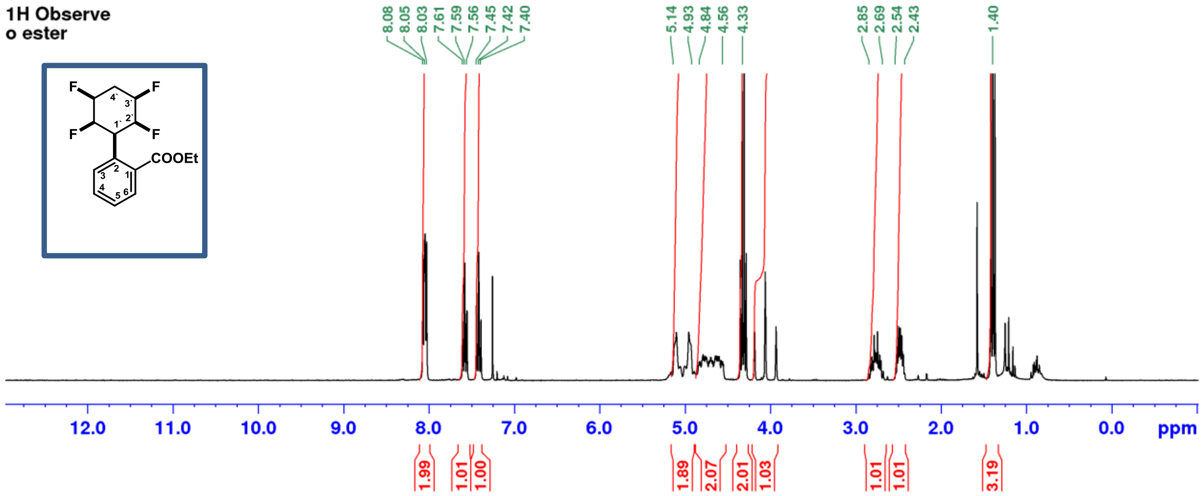

$^{19}F\{^{1}H\}$ NMR of 8 ($CDCl_3$)

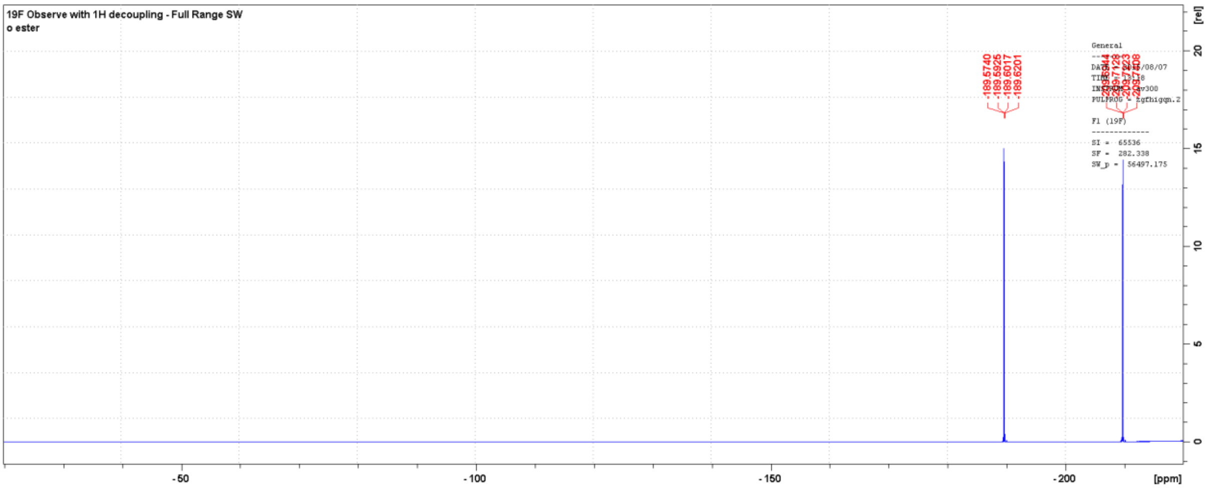

^{13}C NMR of 8 ($CDCl_3$)

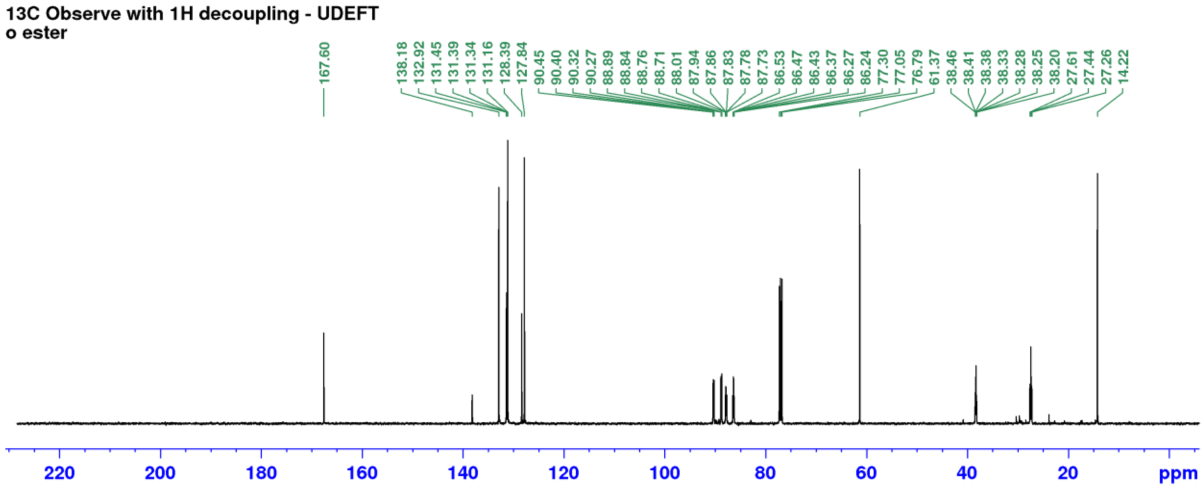

^{1}H NMR of 9 ($CDCl_3$)

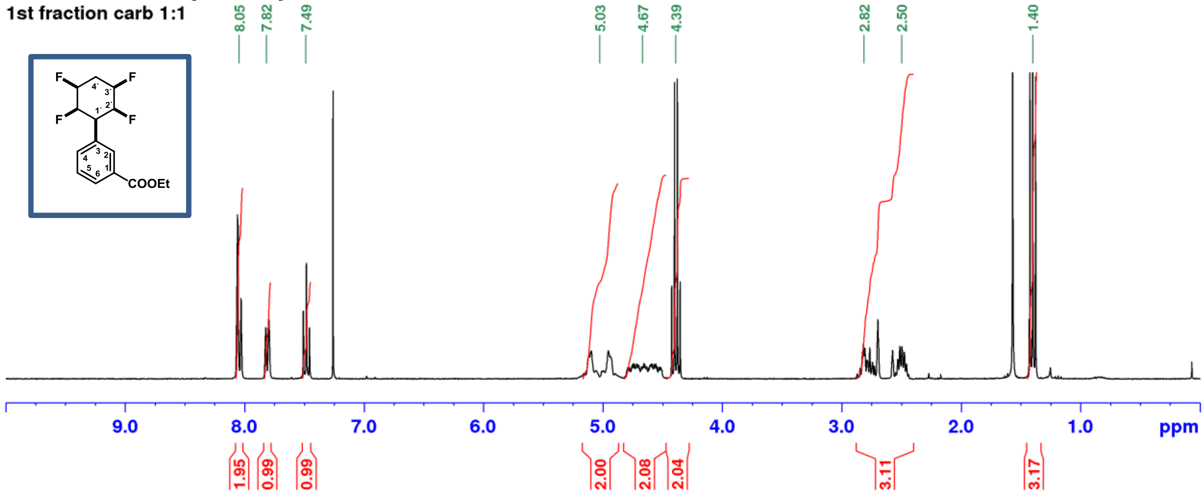

$^{19}F\{^{1}H\}$ NMR of 9 ($CDCl_3$)

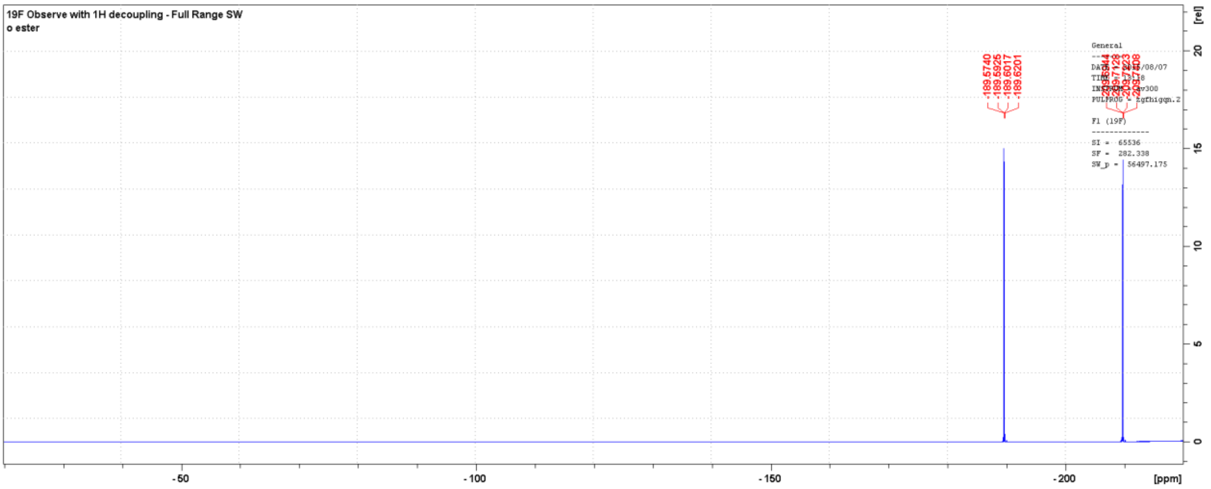

^{13}C NMR of 9 ($CDCl_3$)

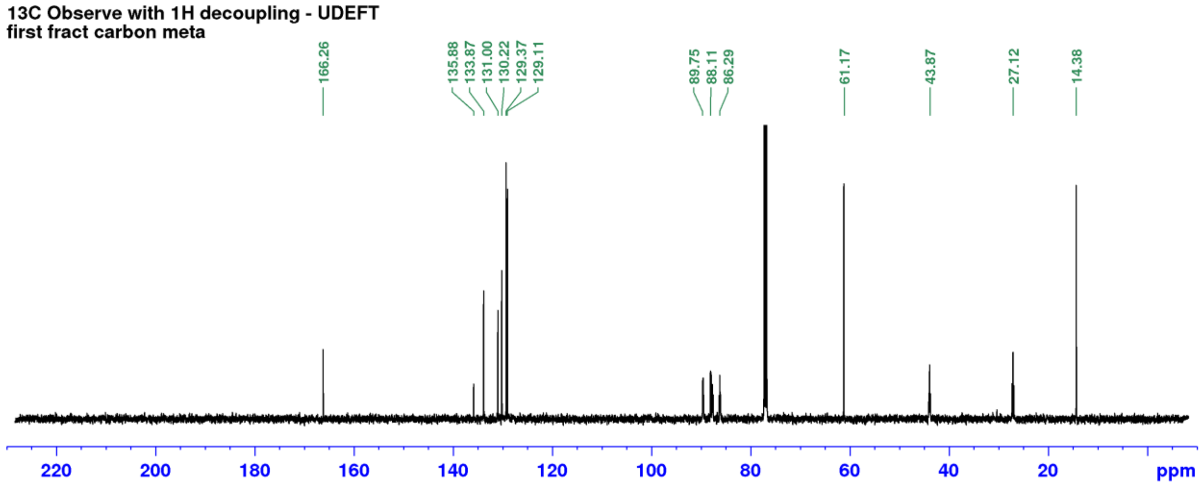

^{1}H NMR of 10 ($CDCl_3$)

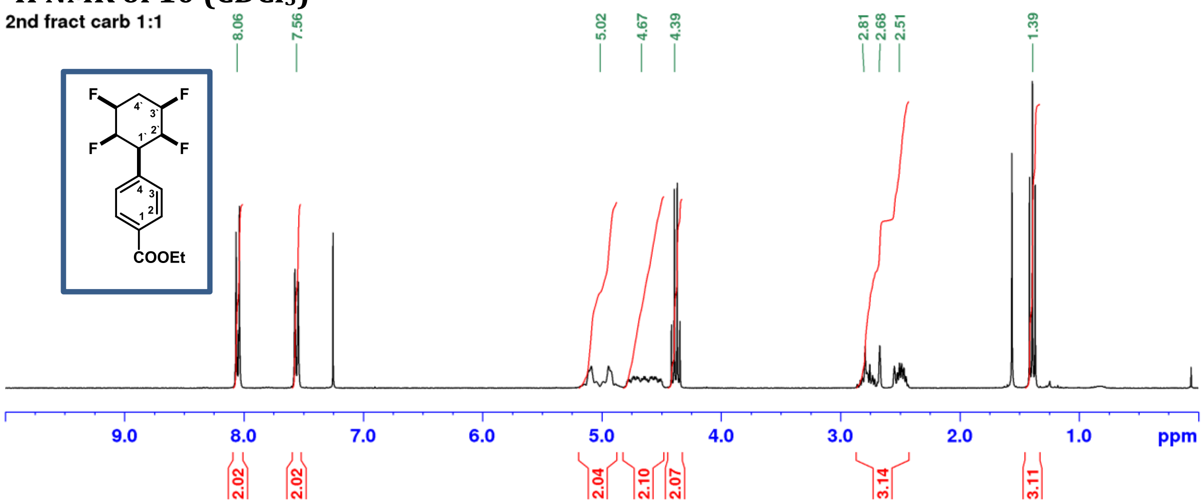

$^{19}F\{^{1}H\}$ NMR of 10 ($CDCl_3$)

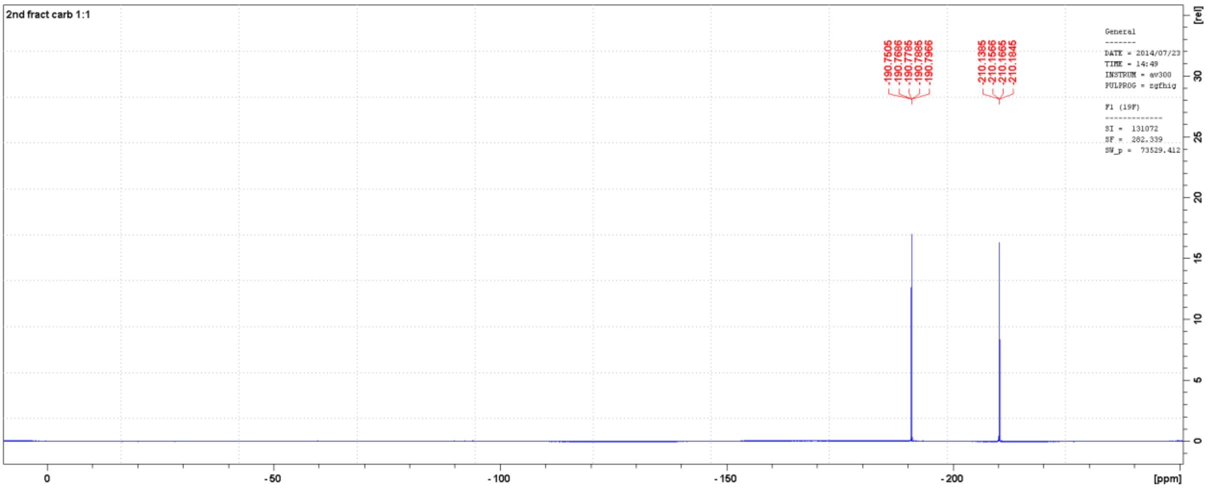

^{13}C NMR of 10 ($CDCl_3$)

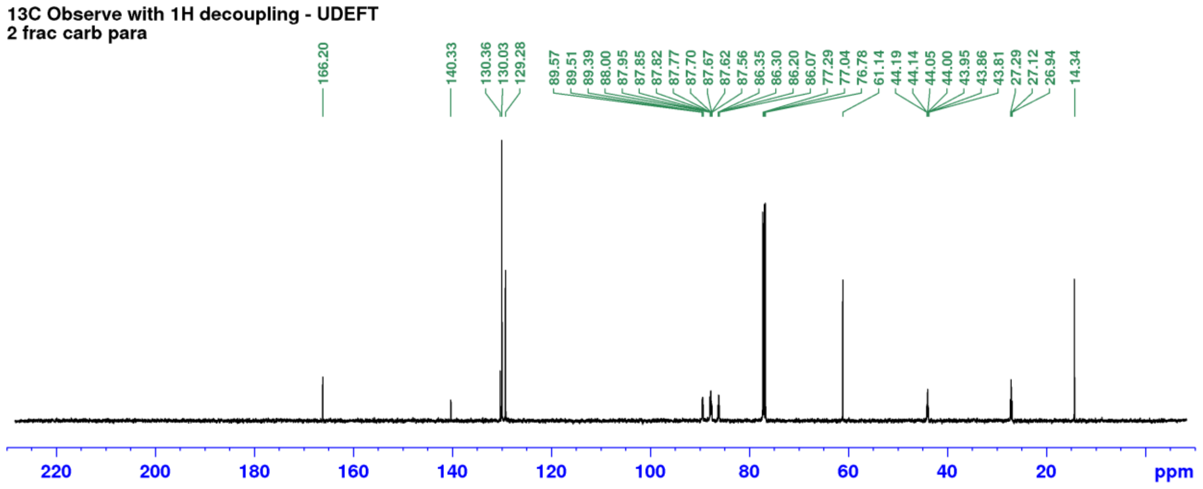

^{1}H NMR of 11 (CD_3COCD_3)

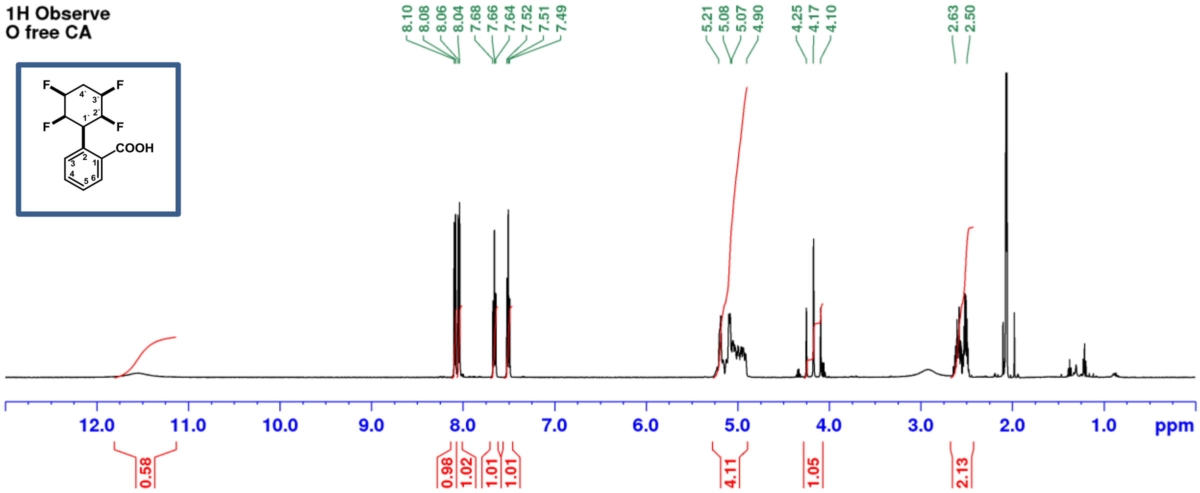

$^{19}F\{^{1}H\}$ NMR of 11 (CD_3COCD_3)

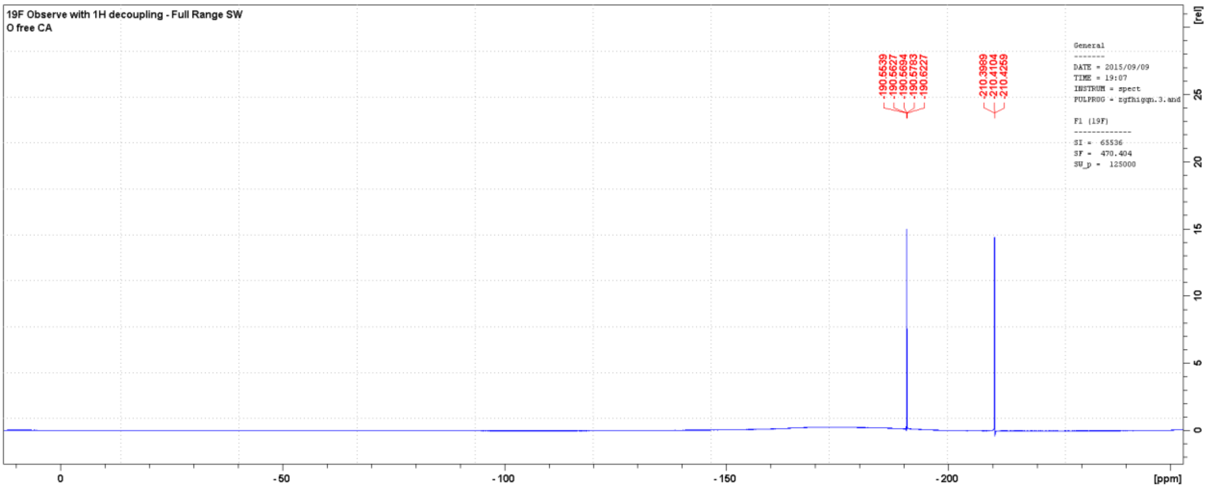

^{13}C NMR of 11 (CD_3COCD_3)

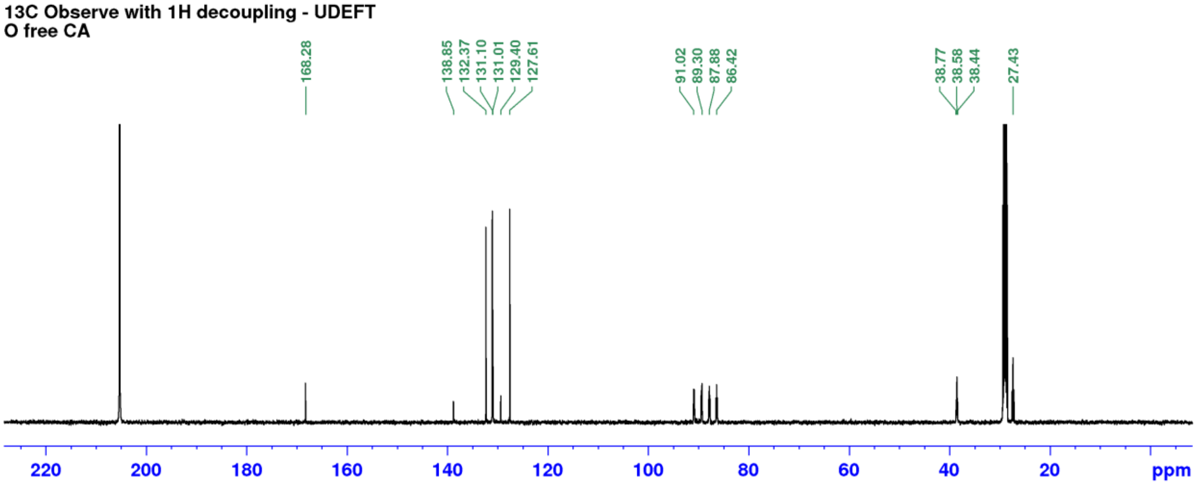

^{1}H NMR of 12 (CD_3COCD_3)

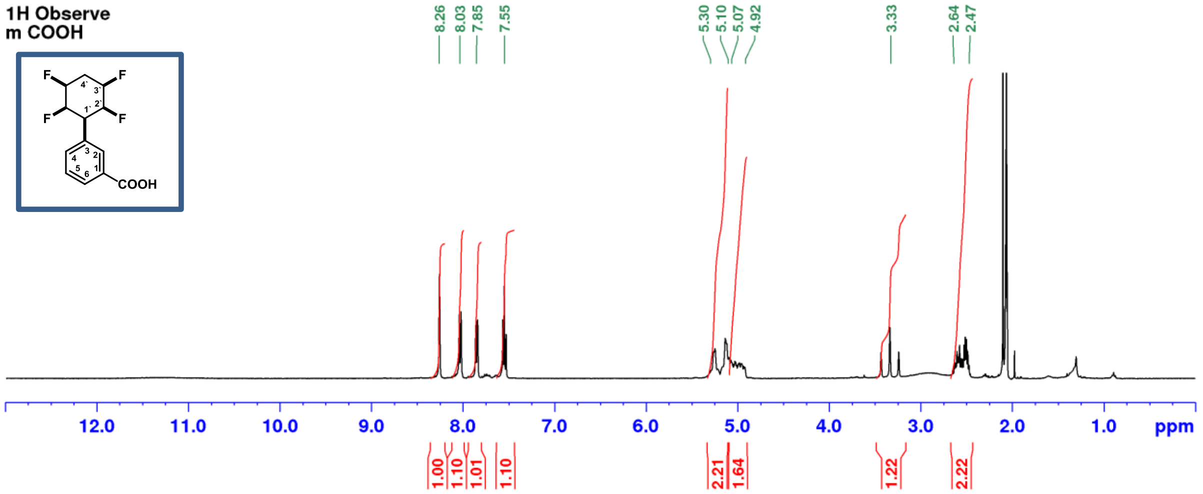

$^{19}F\{^{1}H\}$ NMR of 12 (CD_3COCD_3)

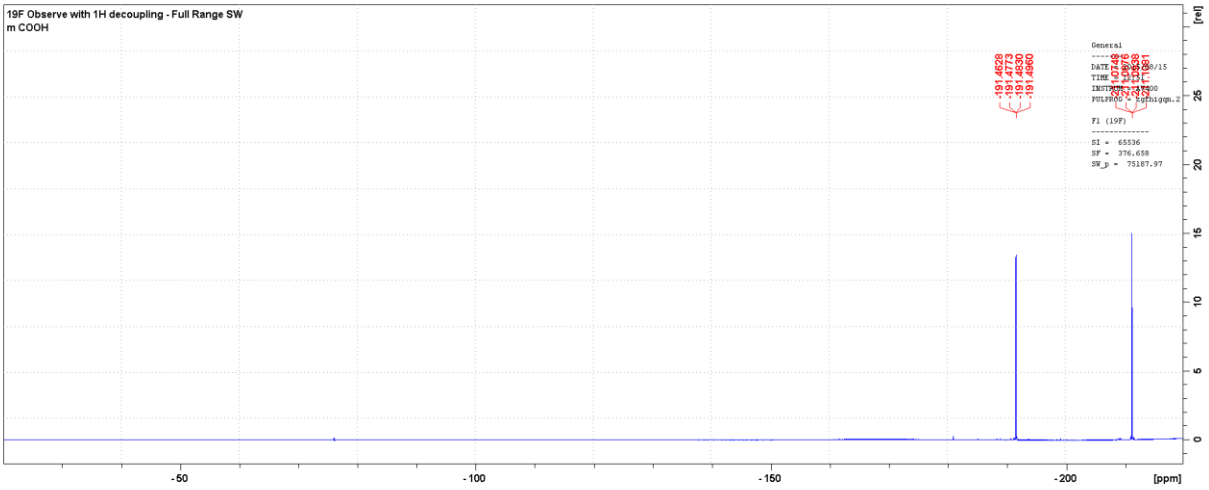

^{13}C NMR of 12 (CD_3COCD_3)

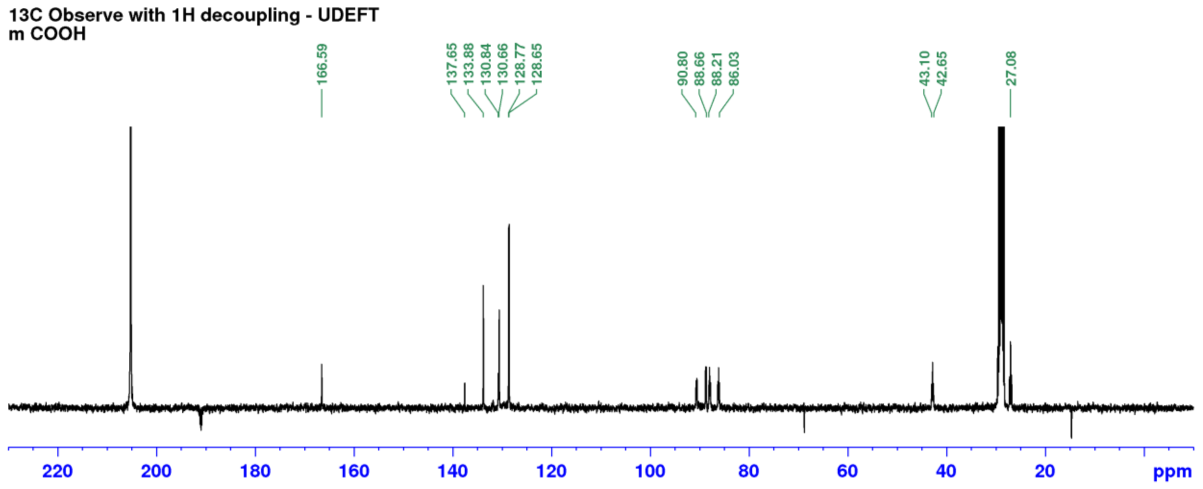

^{1}H NMR of 13 (CD_3COCD_3)

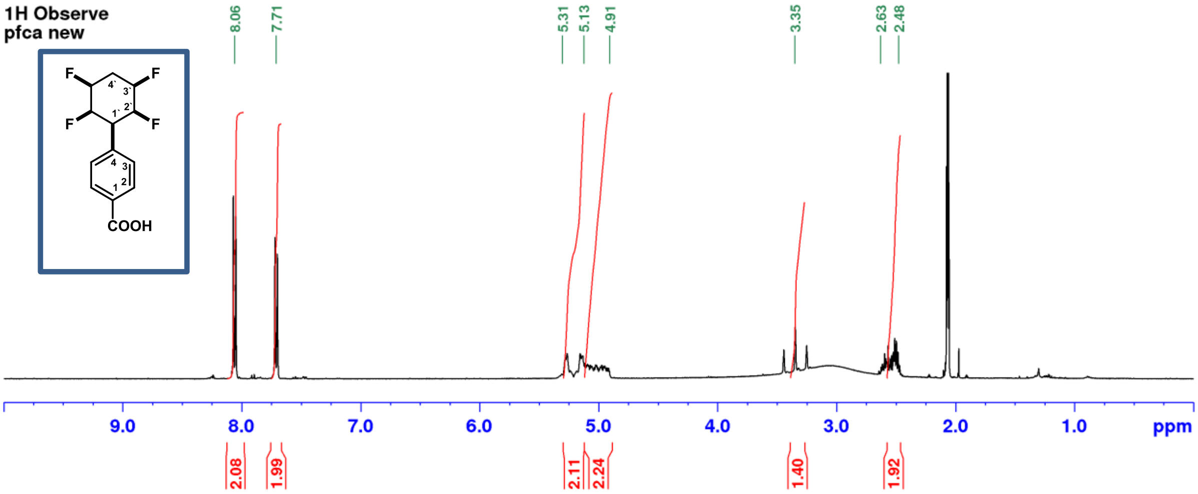

$^{19}F\{^{1}H\}$ NMR of 13 (CD_3COCD_3)

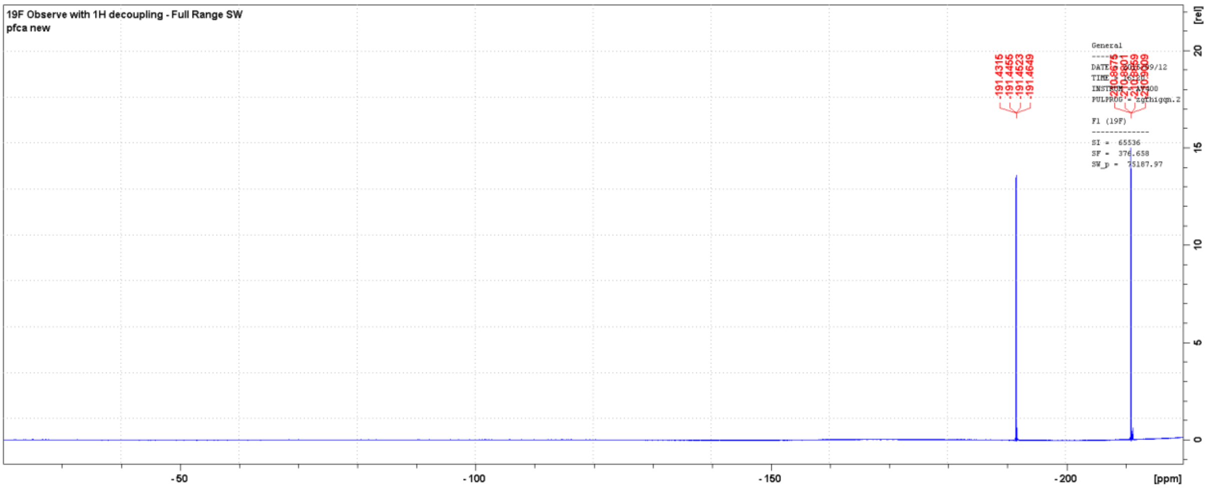

^{13}C NMR of 13 (CD_3COCD_3)

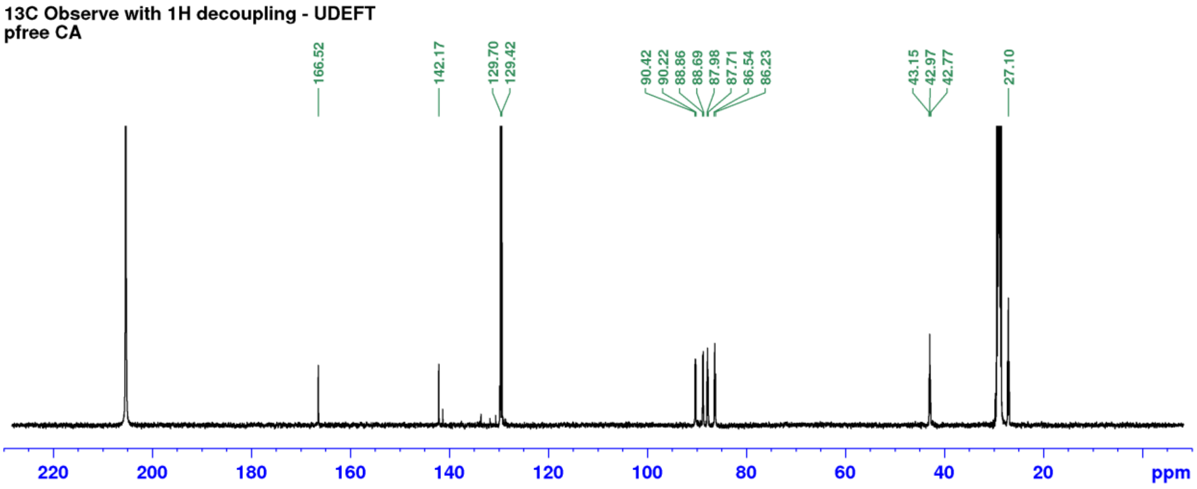

^{1}H NMR of 14 ($CDCl_3$)

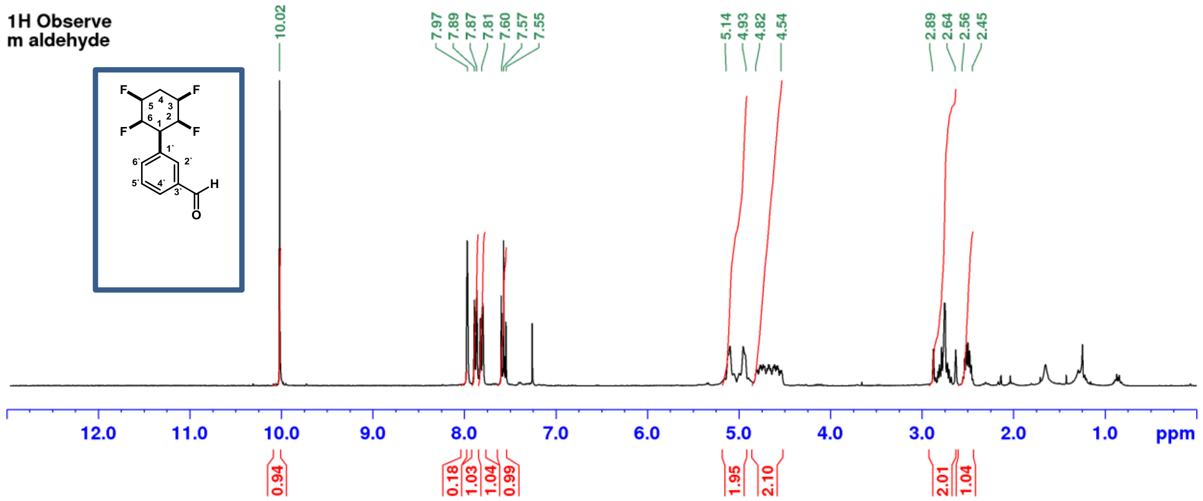

$^{19}F\{^{1}H\}$ NMR of 14 ($CDCl_3$)

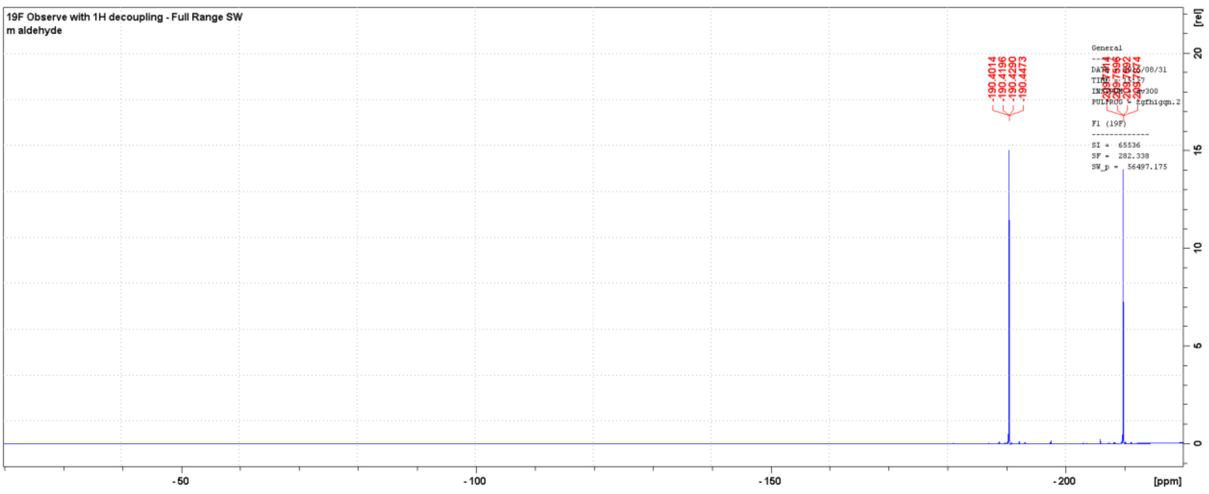

^{13}C NMR of 14 ($CDCl_3$)

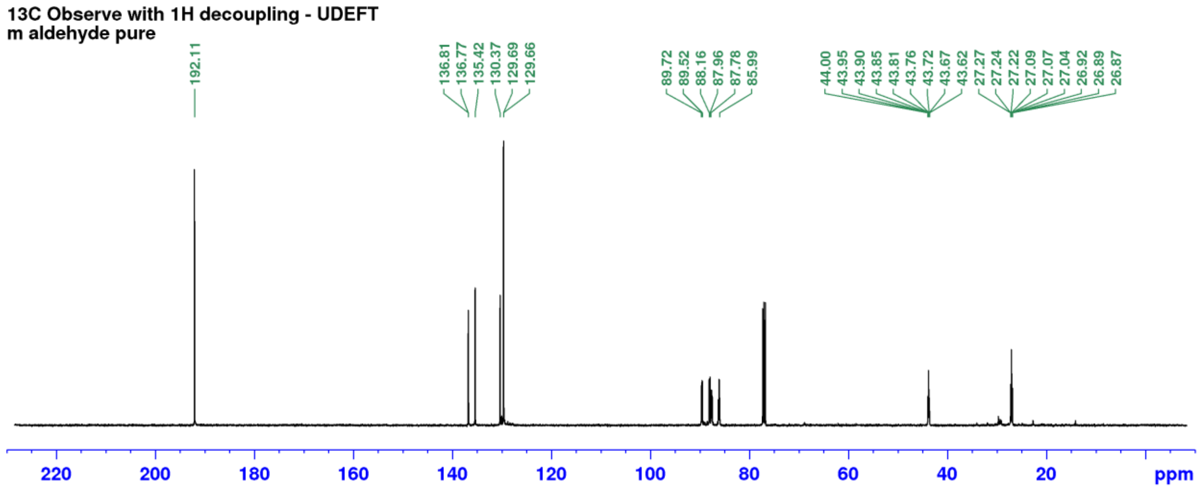

^1H NMR of 15 ($CDCl_3$)

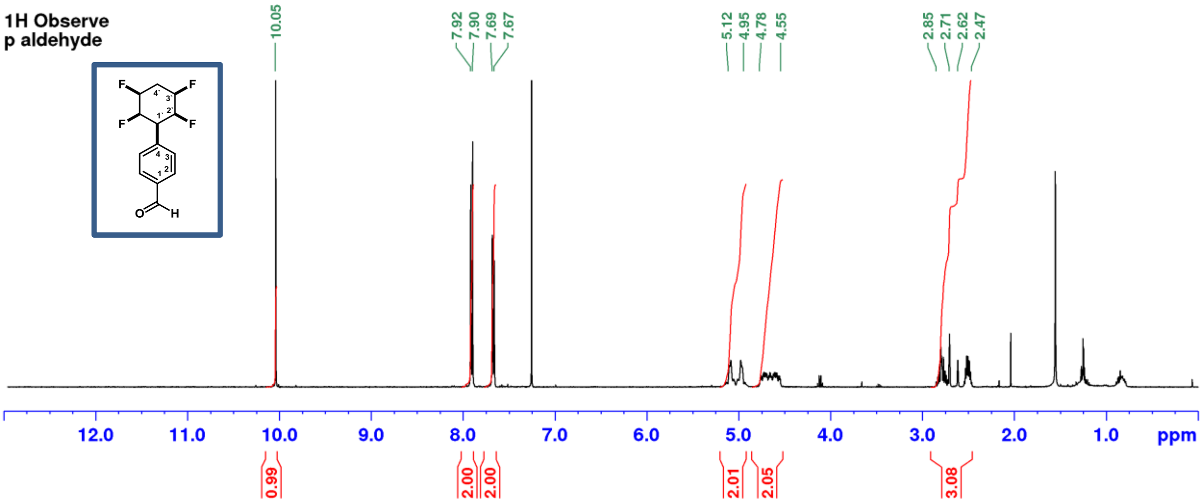

$^{19}F\{^1H\}$ NMR of 15 ($CDCl_3$)

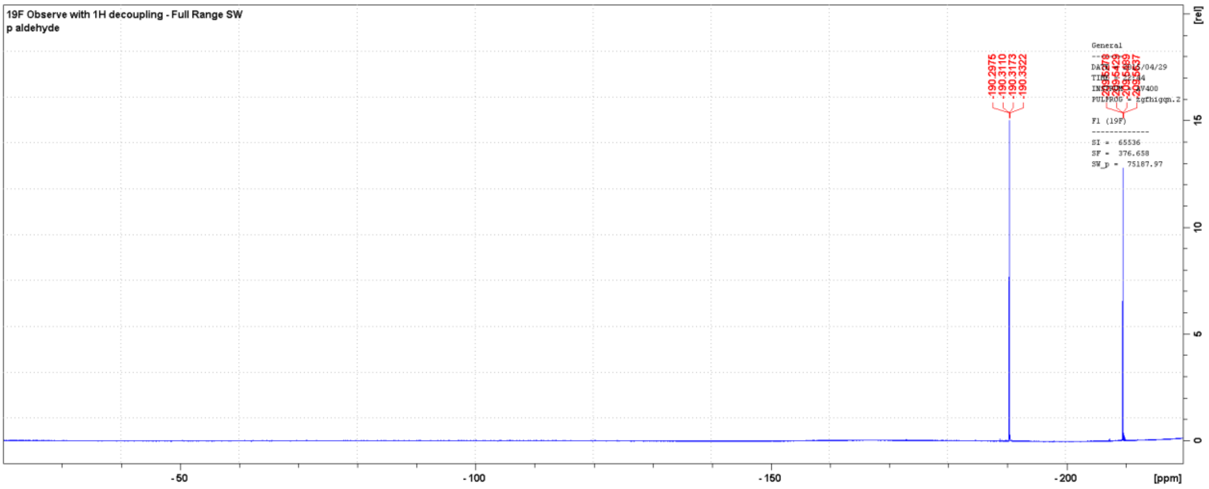

^{13}C NMR of 15 ($CDCl_3$)

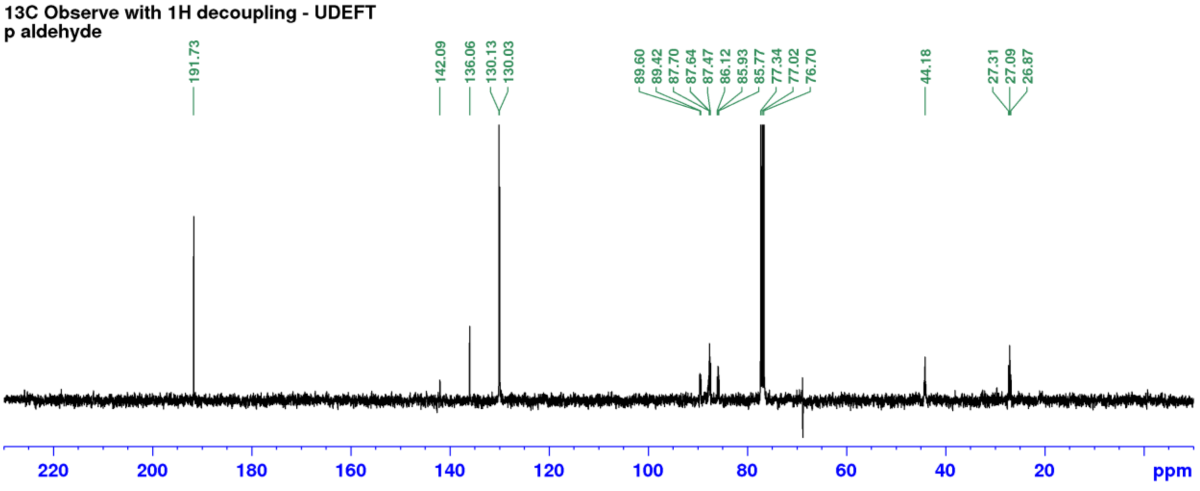

^{1}H NMR of 17 (CD_3COCD_3)

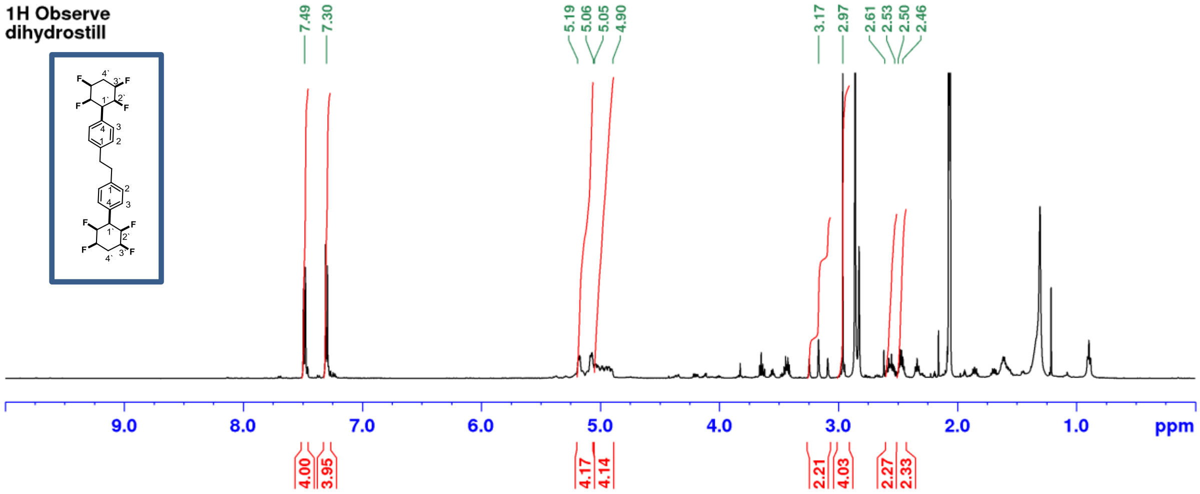

$^{19}F\{^{1}H\}$ NMR of 17 (CD_3COCD_3)

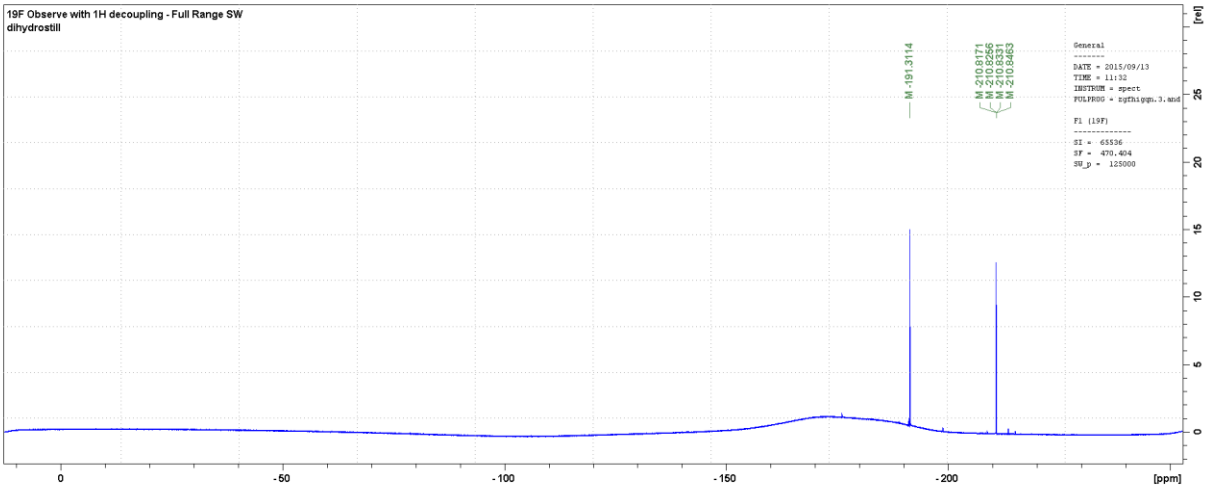

^{13}C NMR of 17 (CD_3COCD_3)

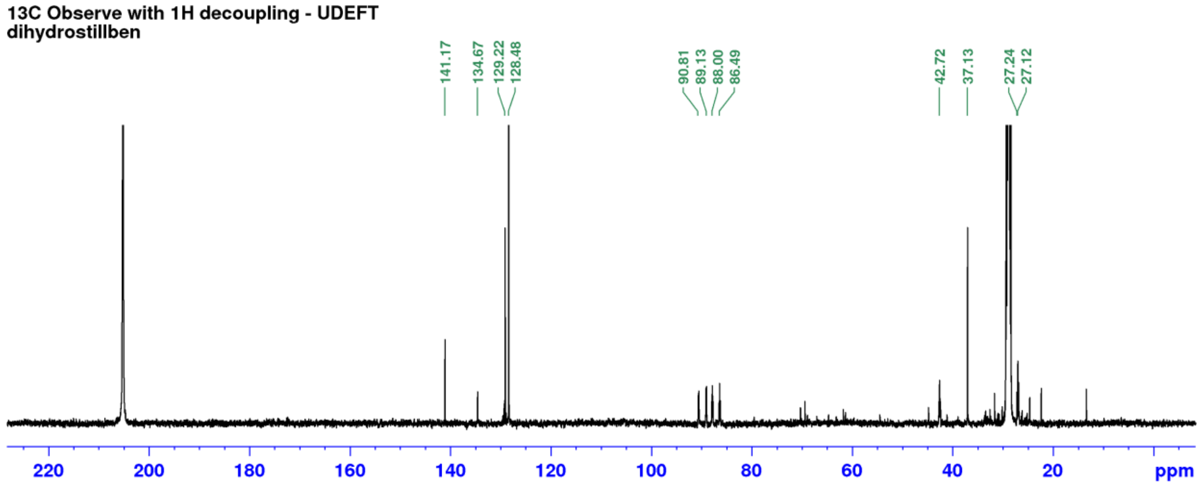

^{1}H NMR of 18 ($CDCl_3$)

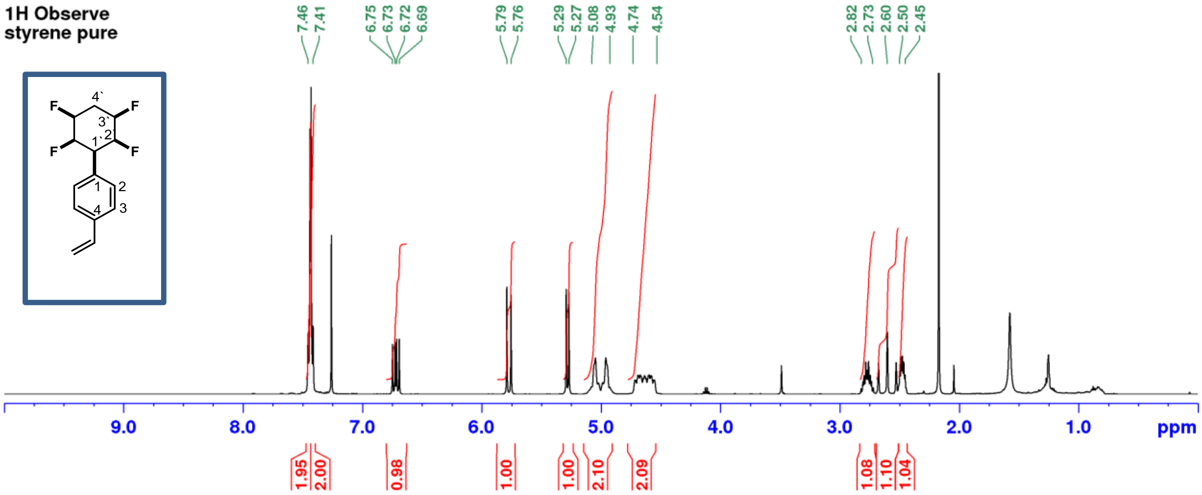

$^{19}F\{^{1}H\}$ NMR of 18 ($CDCl_3$)

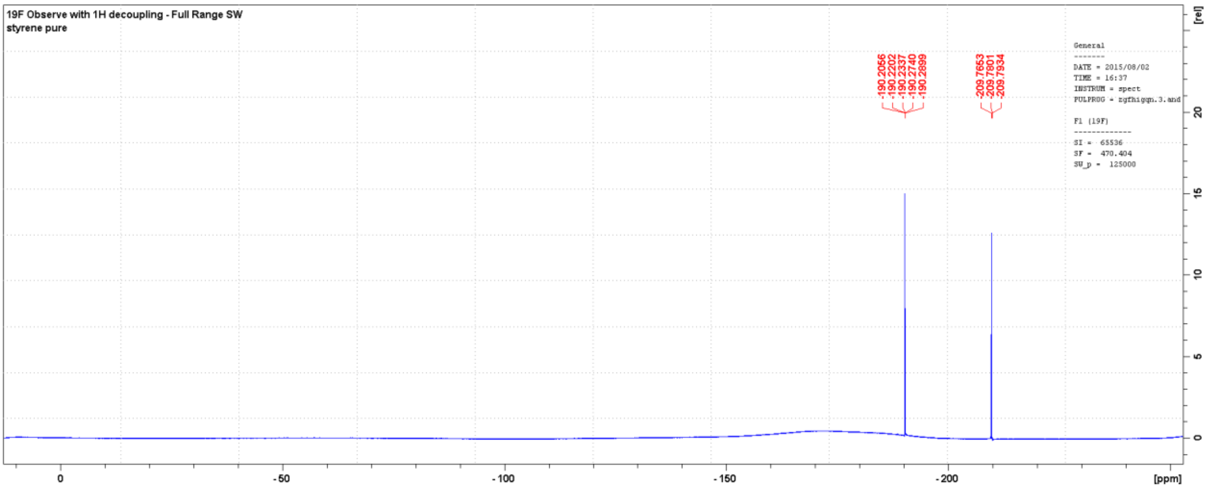

^{13}C NMR of 18 ($CDCl_3$)

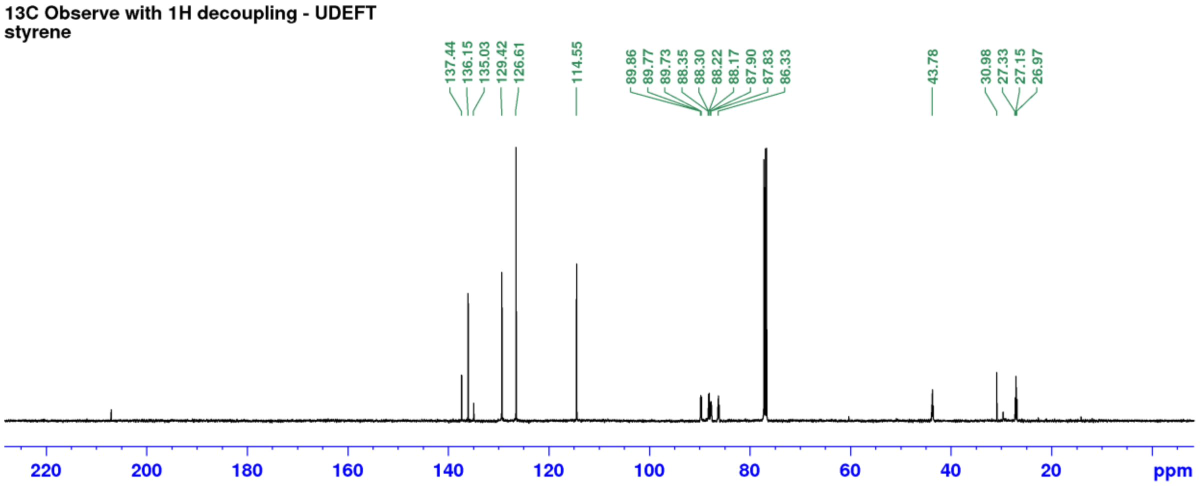

^{1}H NMR of 19 (CD_3COCD_3)

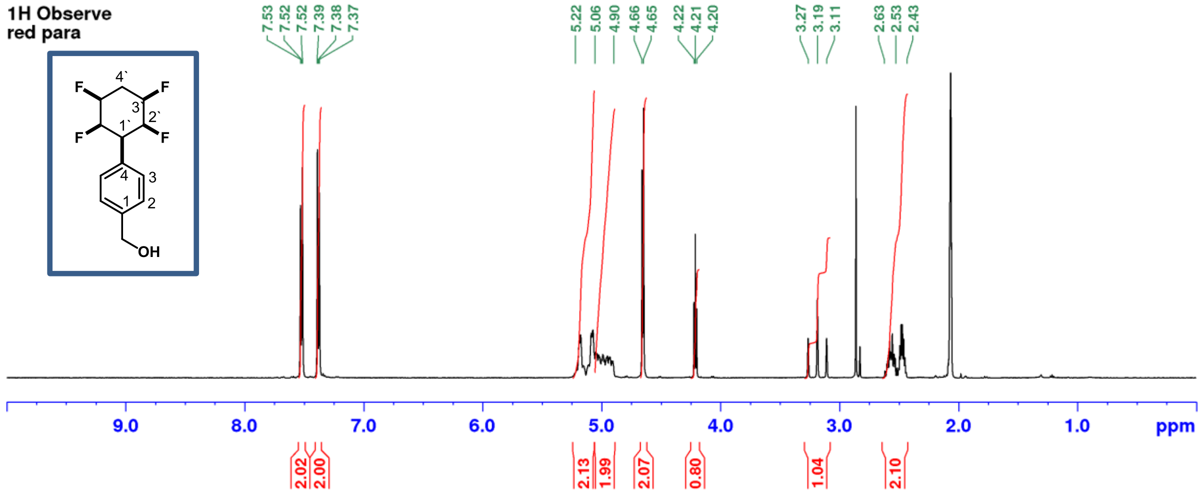

$^{19}F\{^{1}H\}$ NMR of 19 (CD_3COCD_3)

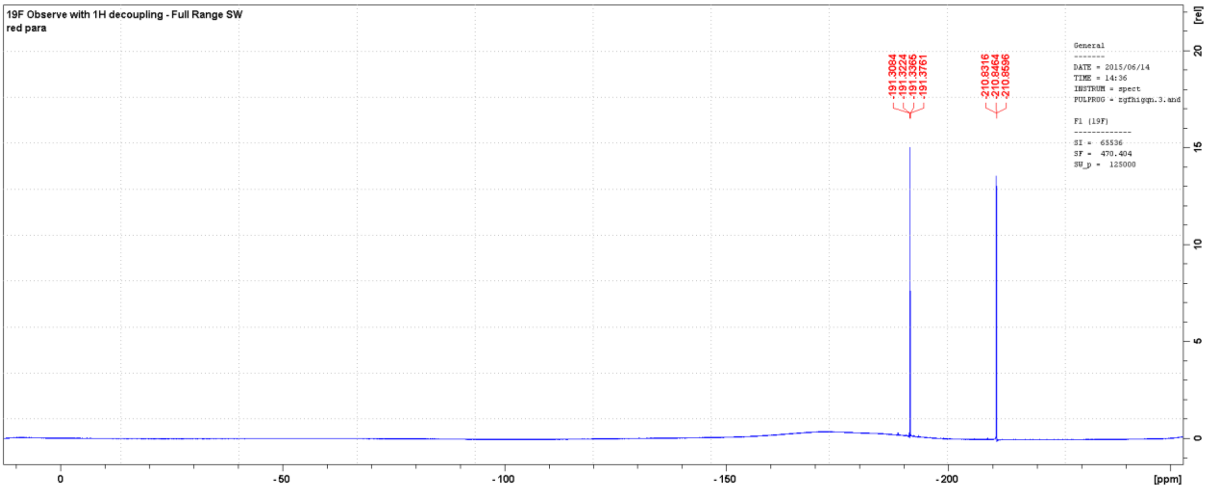

^{13}C NMR of 19 (CD_3COCD_3)

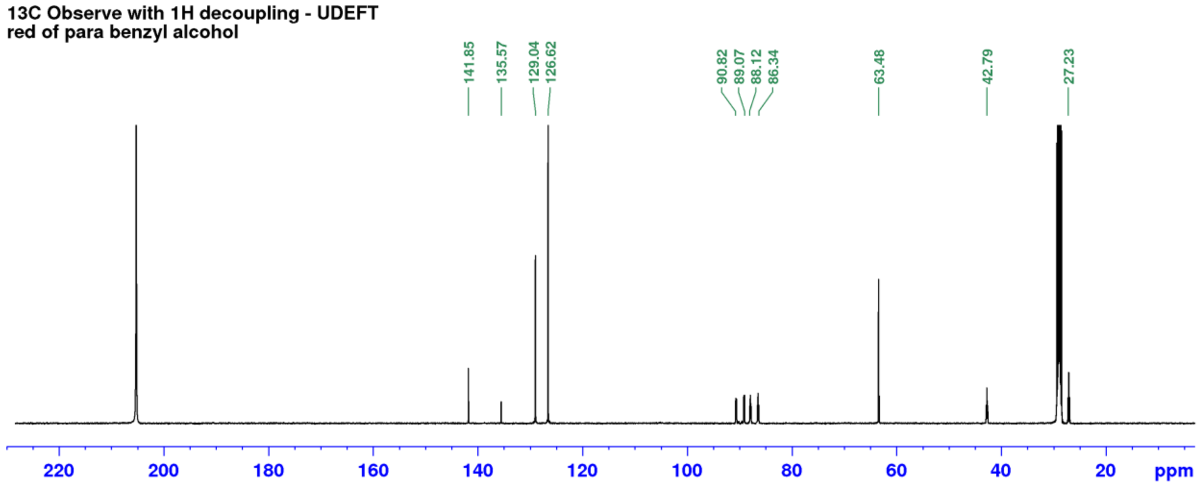

^{1}H NMR of 20 ($CDCl_3$)

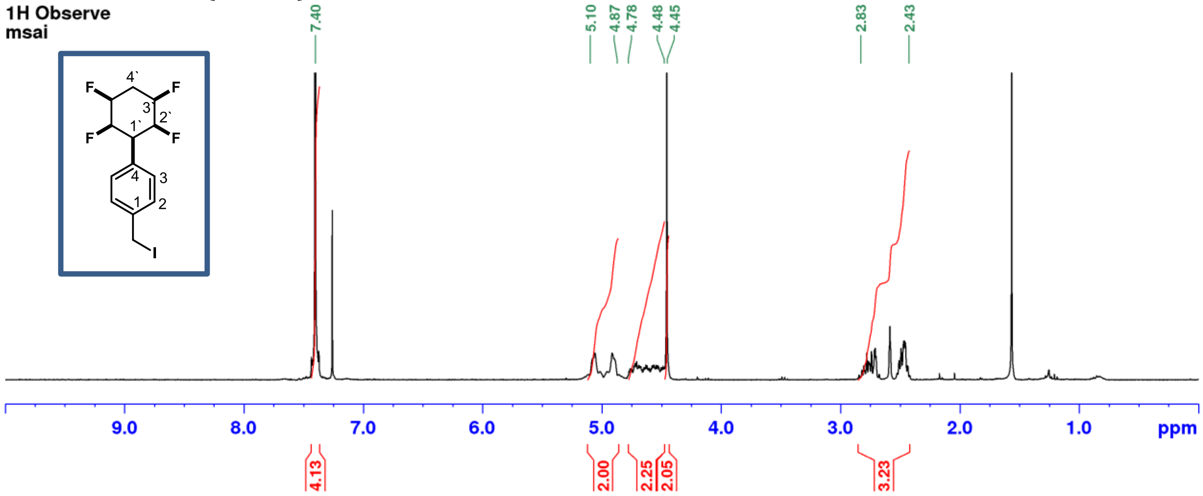

$^{19}F\{^{1}H\}$ NMR of 20 ($CDCl_3$)

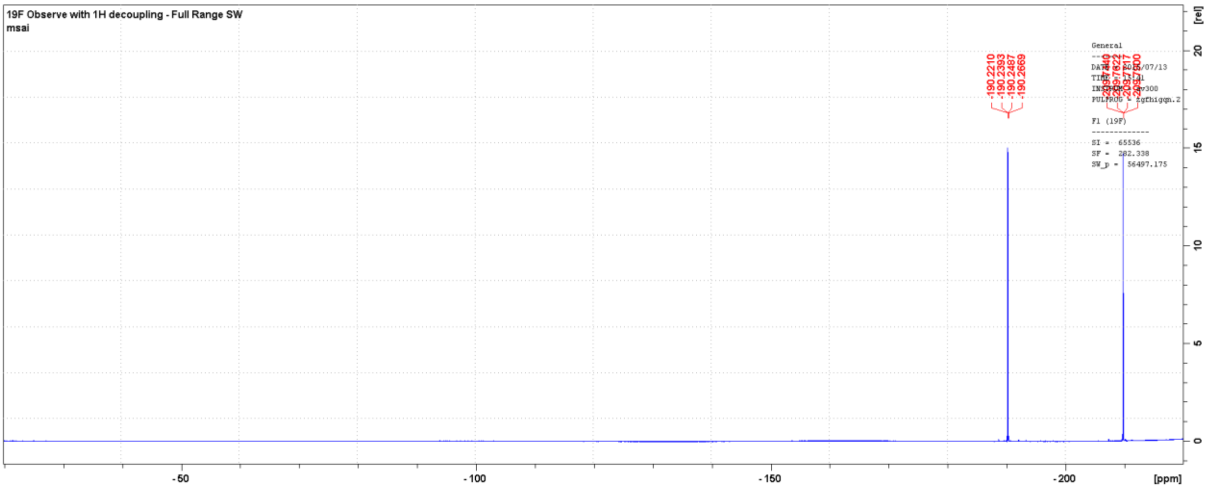

^{13}C NMR of 20 ($CDCl_3$)

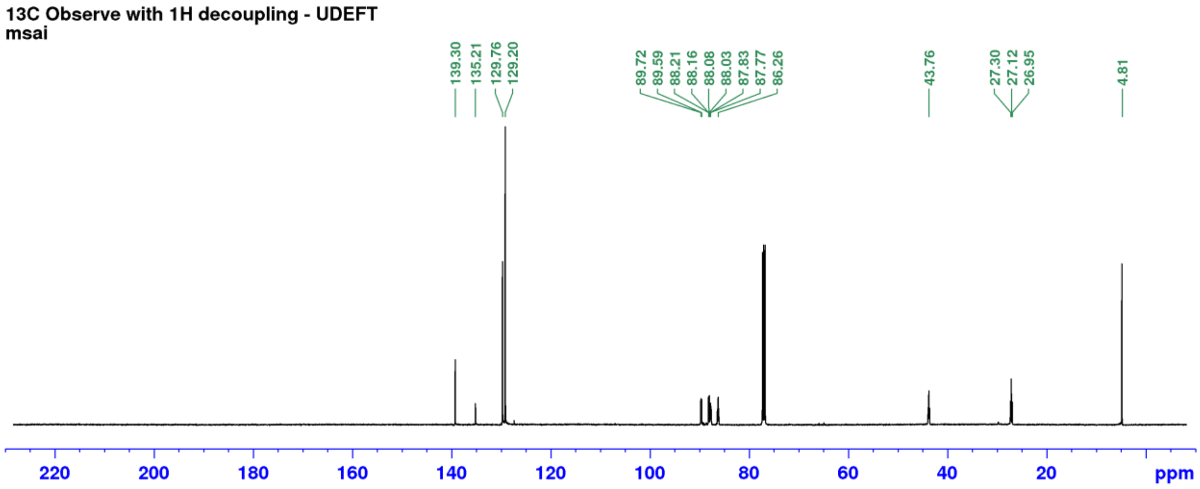

^{1}H NMR of 21 ($CDCl_3$)

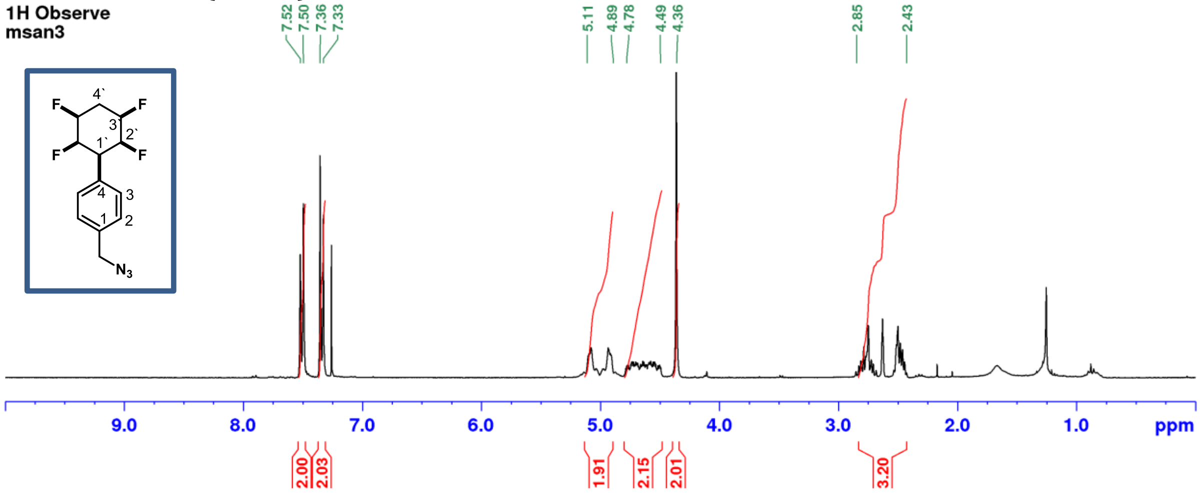

$^{19}F\{^{1}H\}$ NMR of 21 ($CDCl_3$)

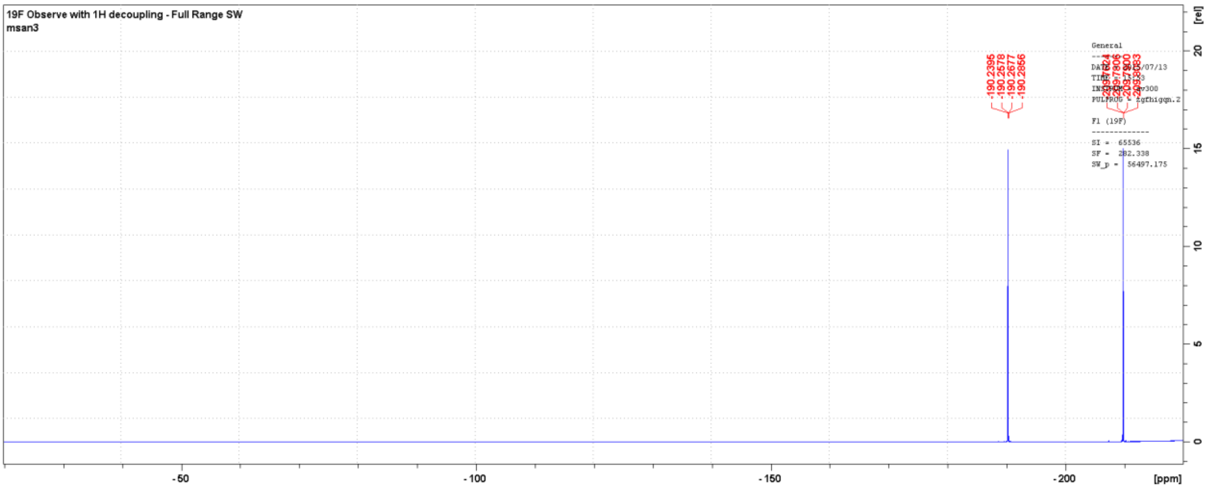

^{13}C NMR of 21 ($CDCl_3$)

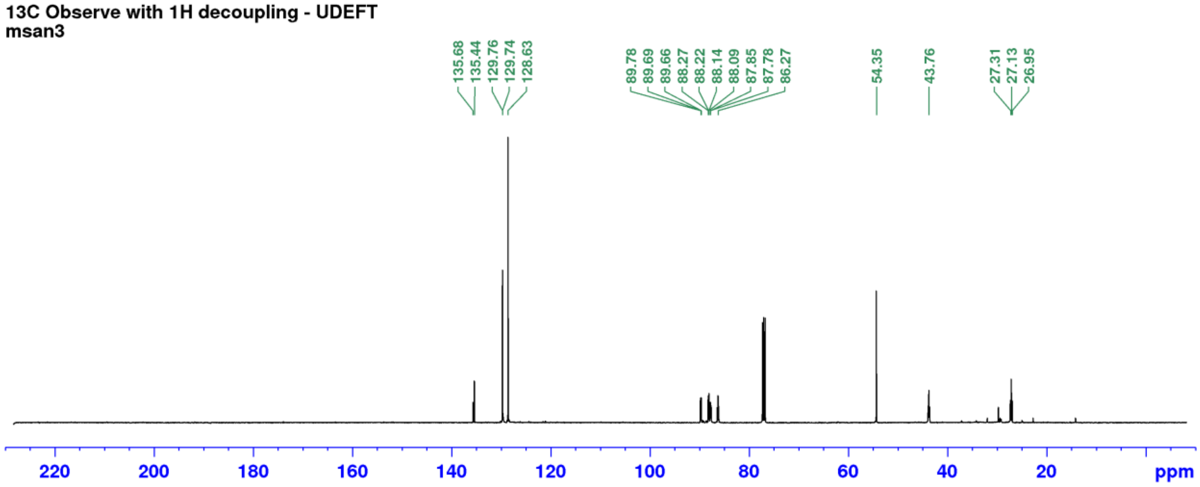

^{1}H NMR of 23 ($CDCl_3$)

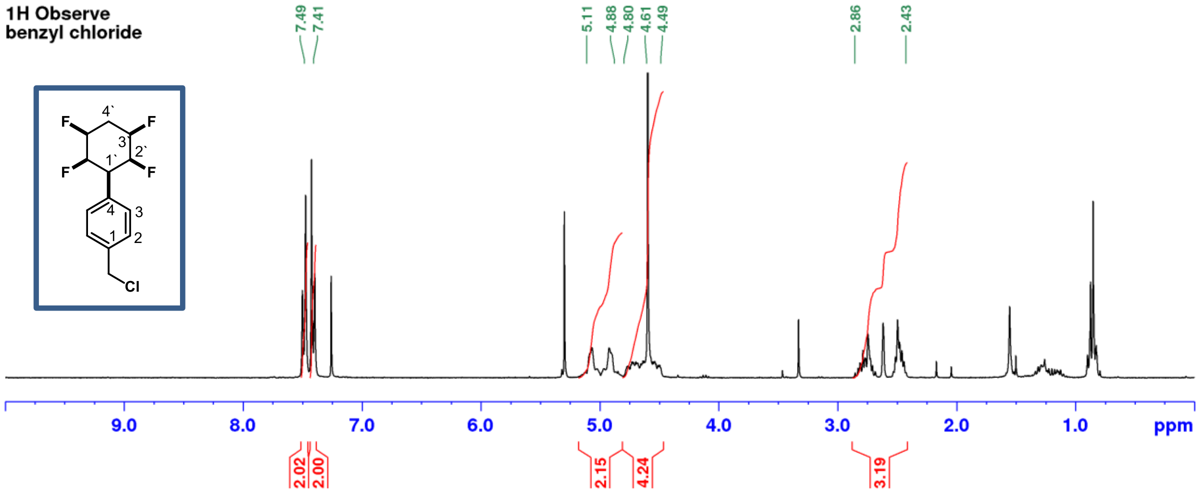

$^{19}F\{^{1}H\}$ NMR of 23 ($CDCl_3$)

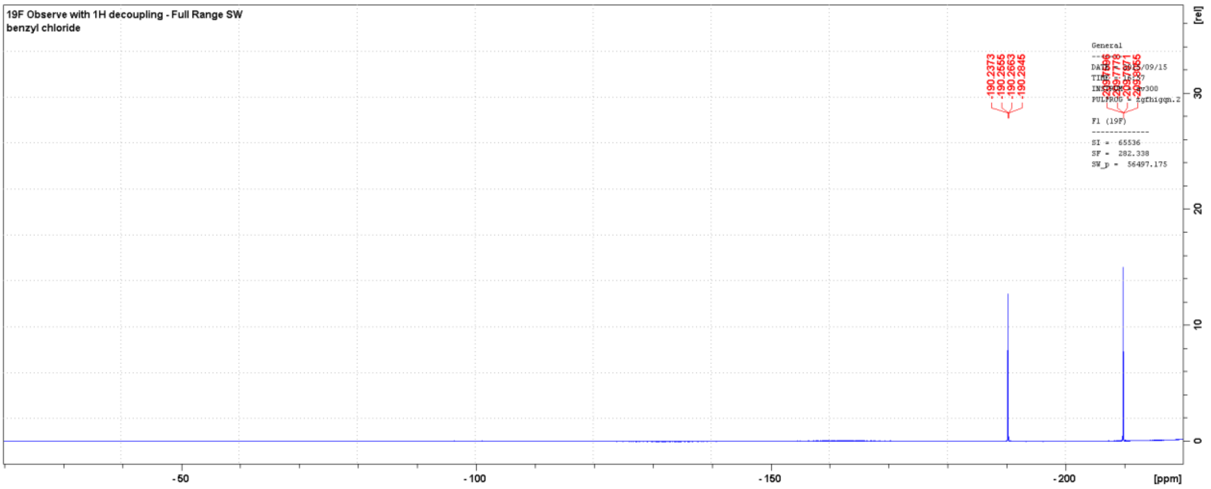

^{13}C NMR of 23 ($CDCl_3$)

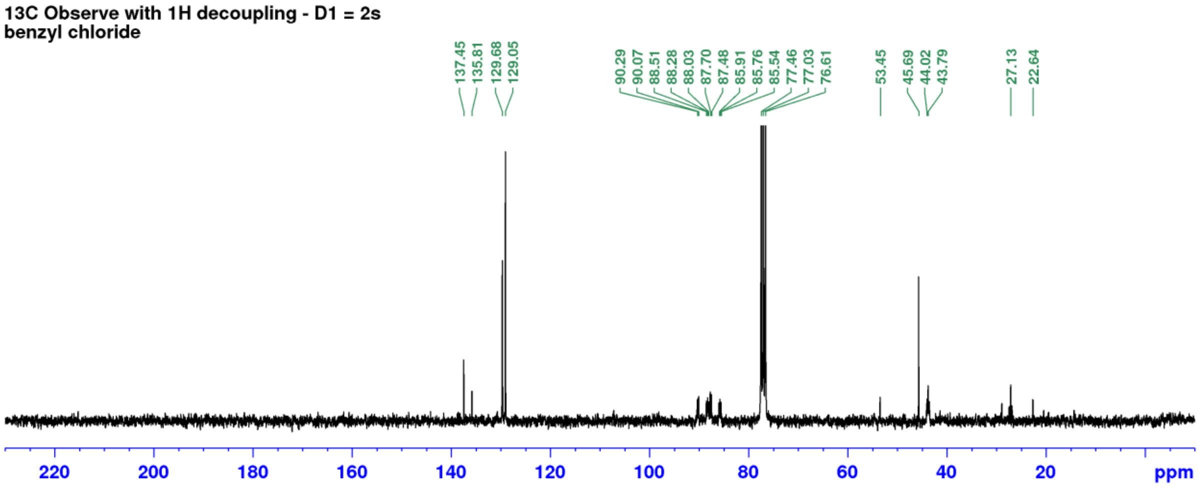

^{1}H NMR of 25 ($CDCl_3$)

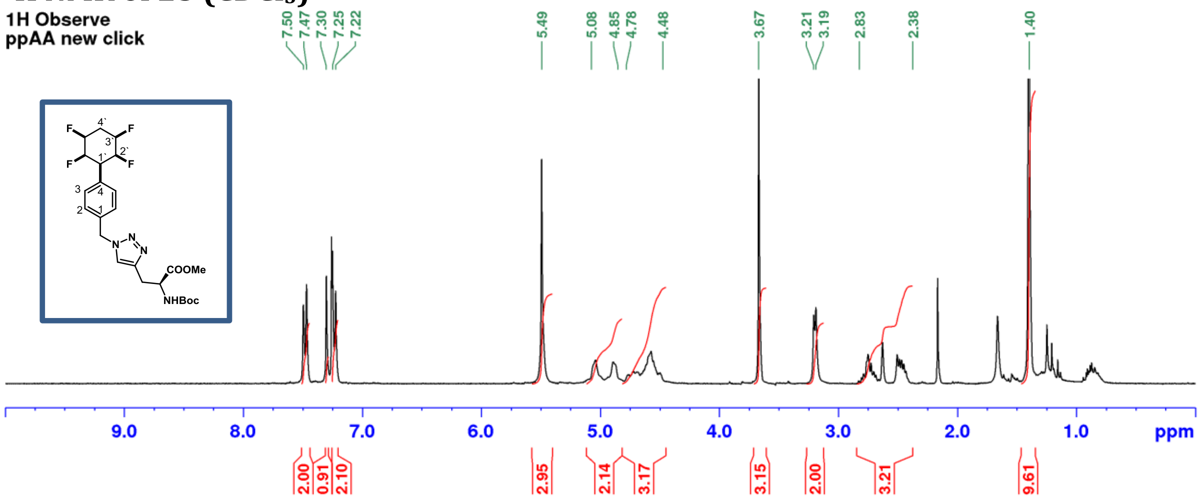

$^{19}F\{^{1}H\}$ NMR of 25 ($CDCl_3$)

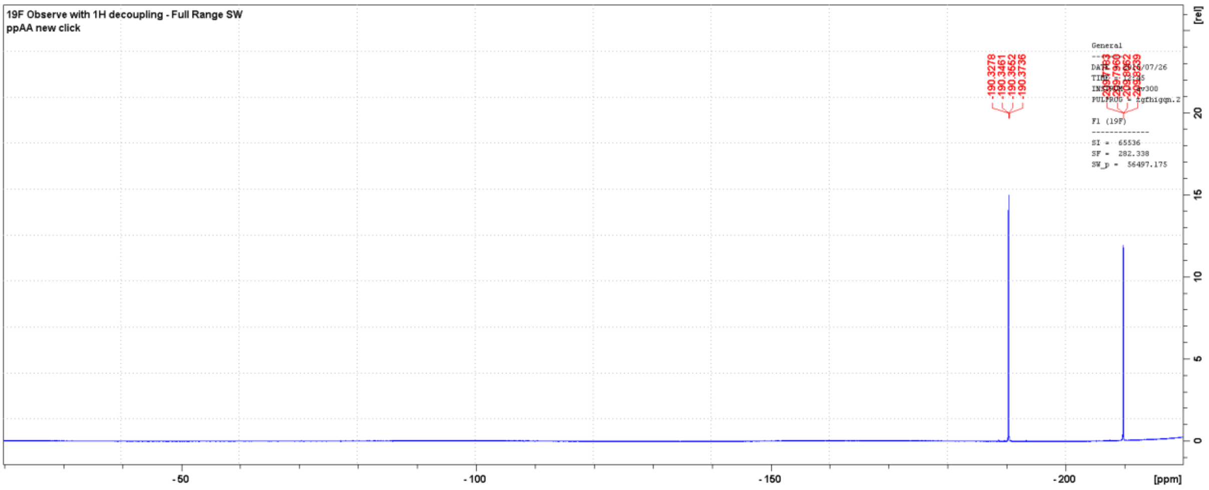

^{13}C NMR of 25 ($CDCl_3$)

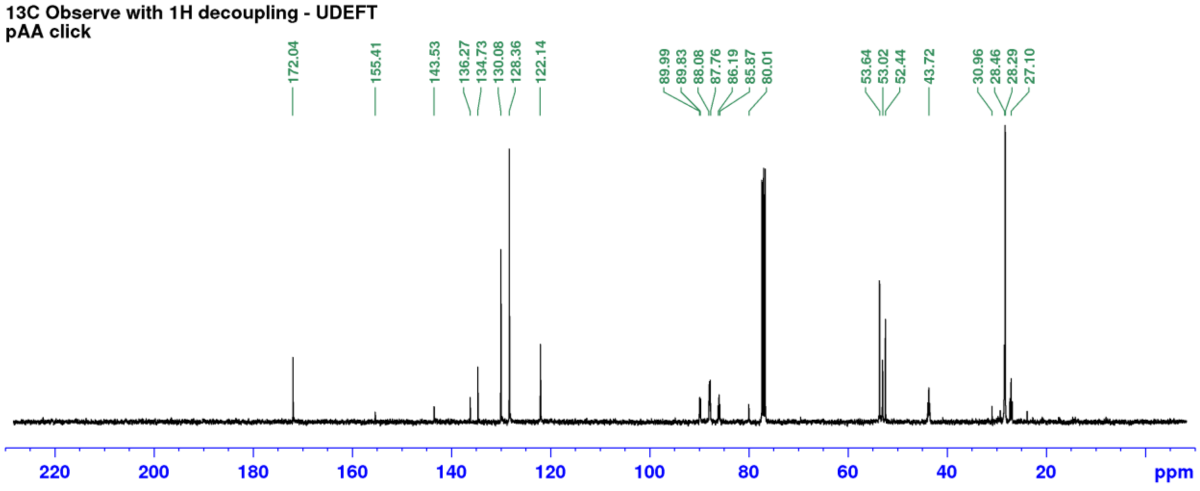

^{1}H NMR of 26 (d_6-DMSO)

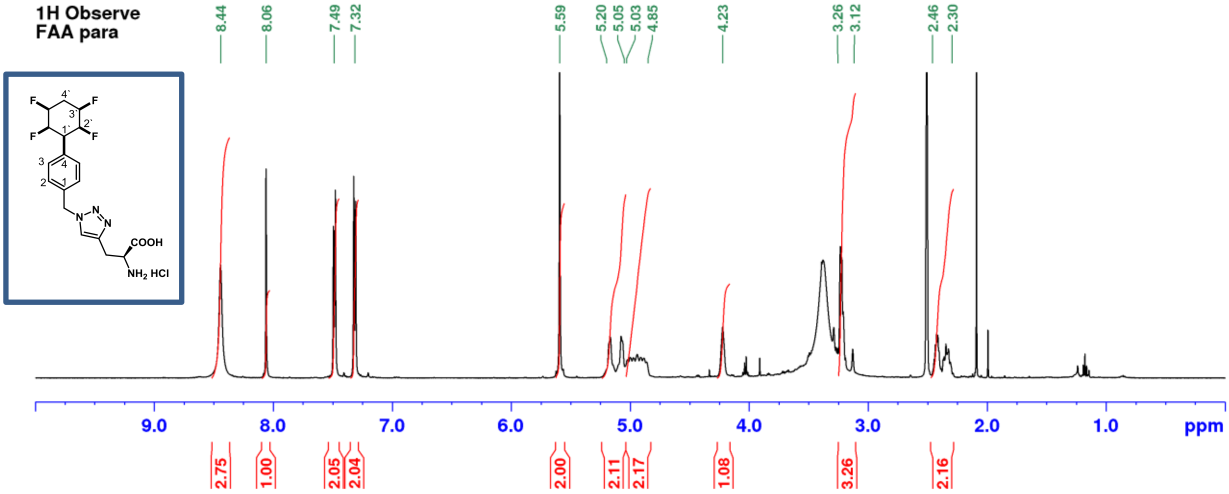

^{19}F{^{1}H} NMR of 26 (d_6-DMSO)

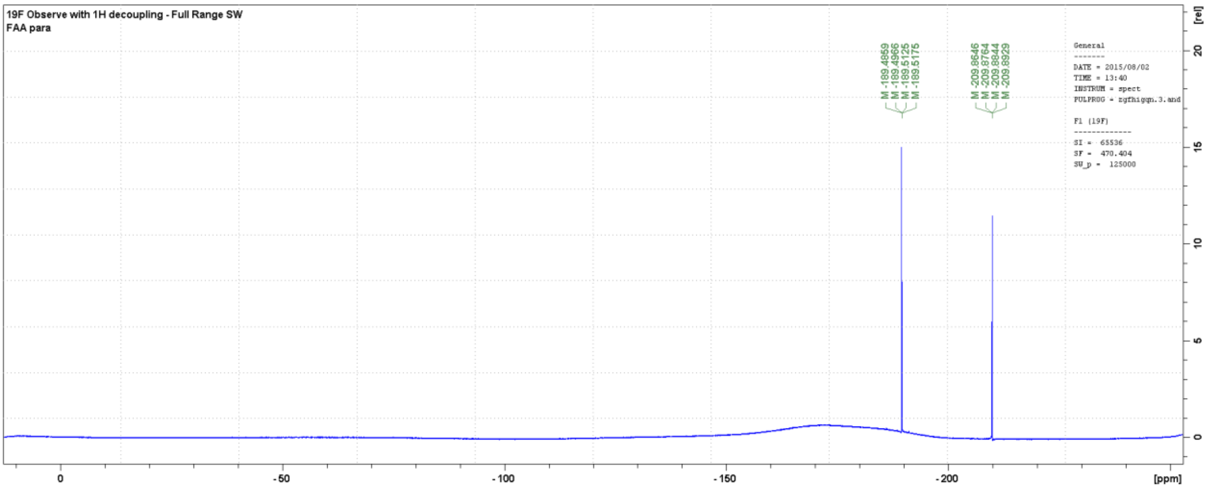

^{13}C NMR of 26 (d_6-DMSO)

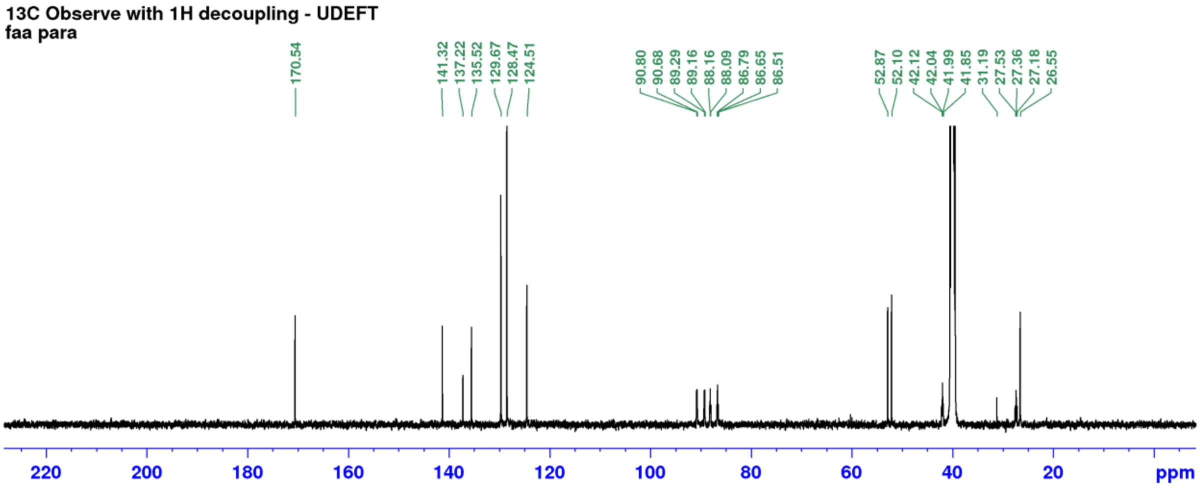

^{1}H NMR of 27 (d_6-DMSO)

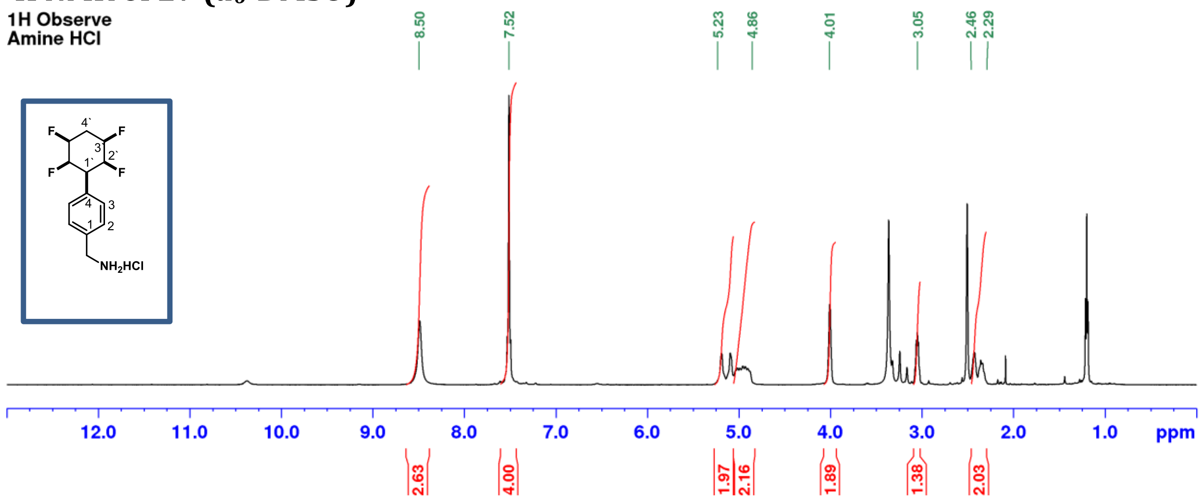

$^{19}F\{^{1}H\}$ NMR of 27 (d_6-DMSO)

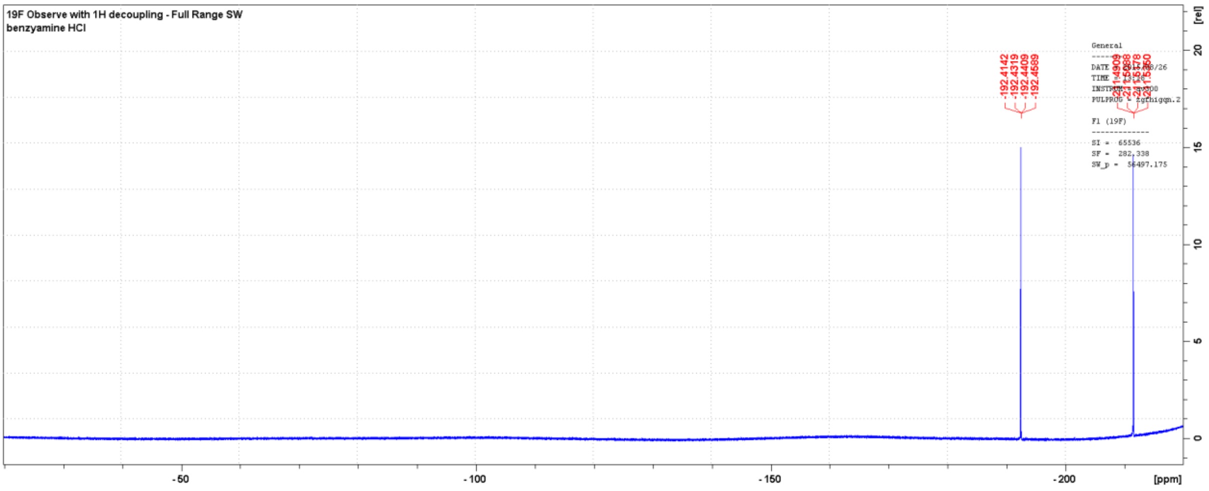

^{13}C NMR of 27 (d_6-DMSO)

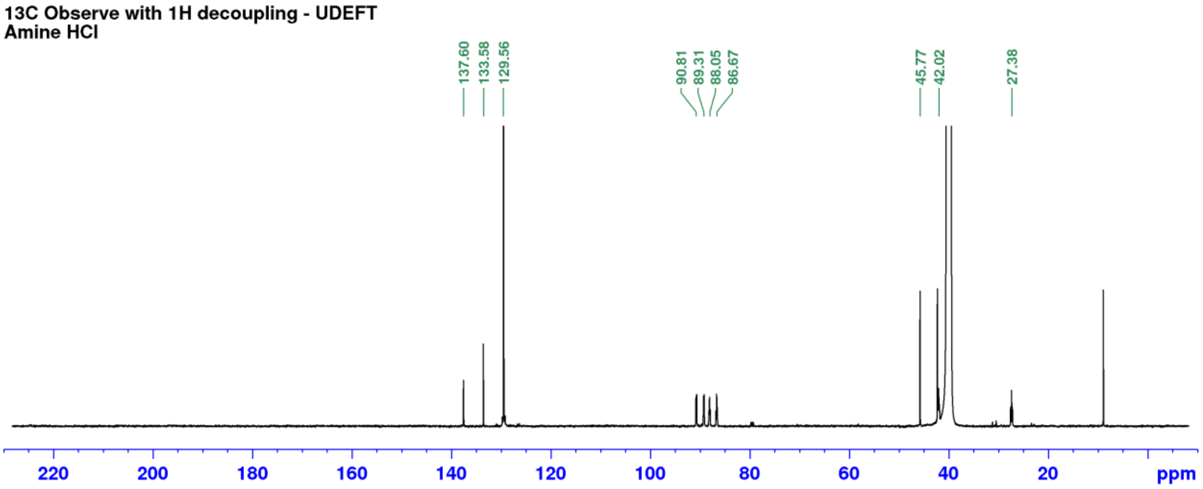

^{1}H NMR of 28 ($CDCl_3$)

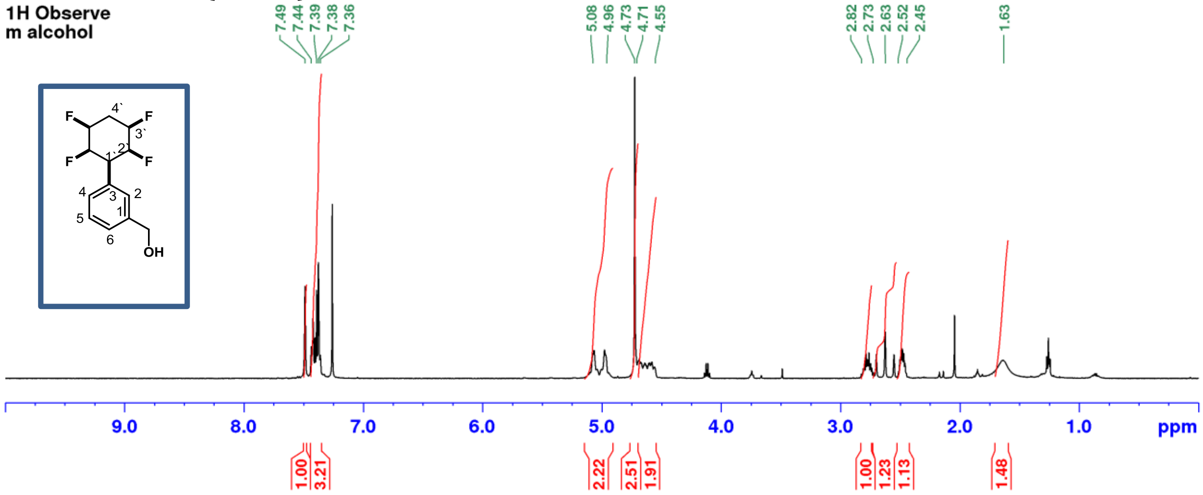

$^{19}F\{^{1}H\}$ NMR of 28 ($CDCl_3$)

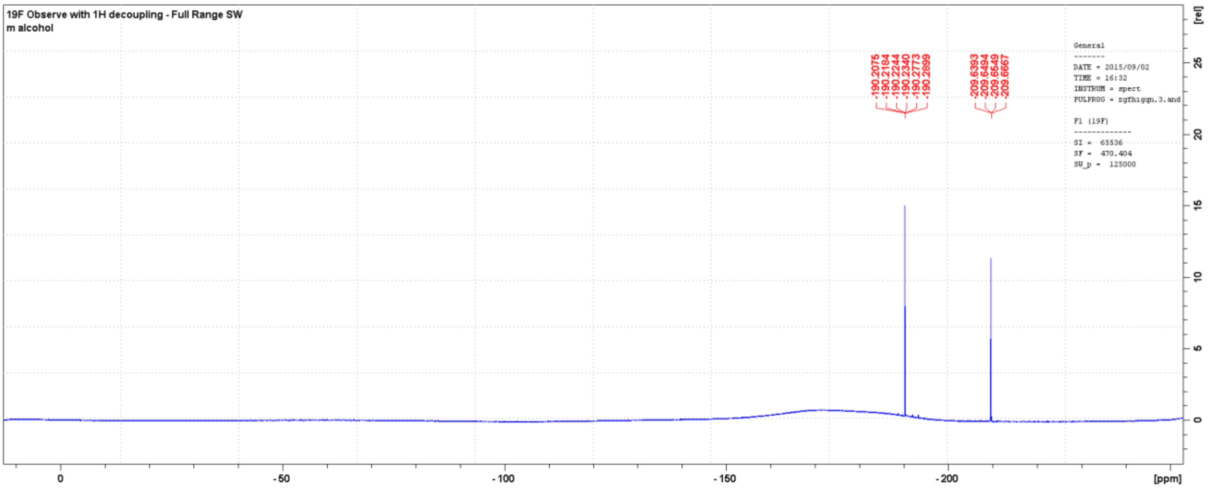

^{13}C NMR of 28 ($CDCl_3$)

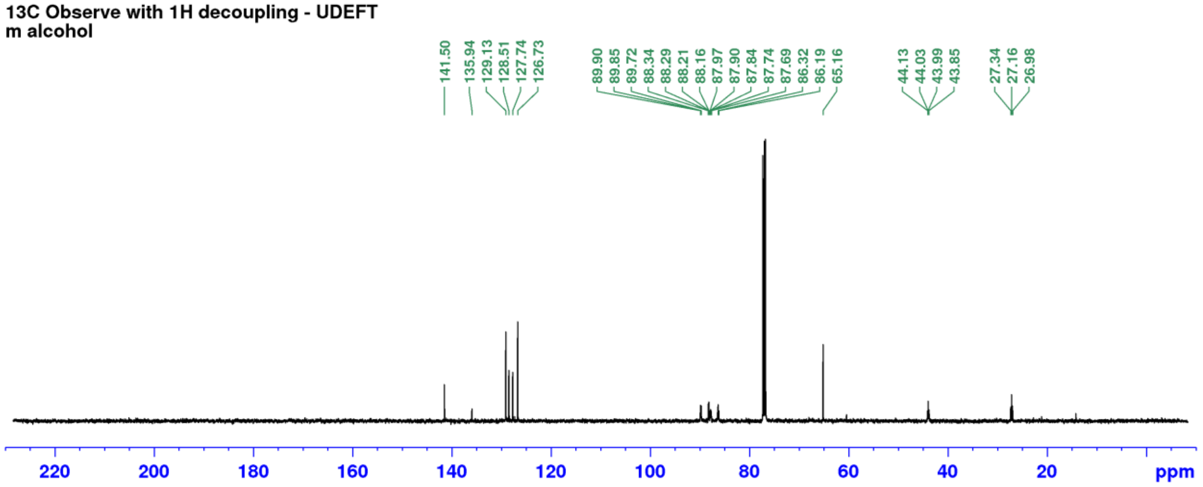

^{1}H NMR of 29 ($CDCl_3$)

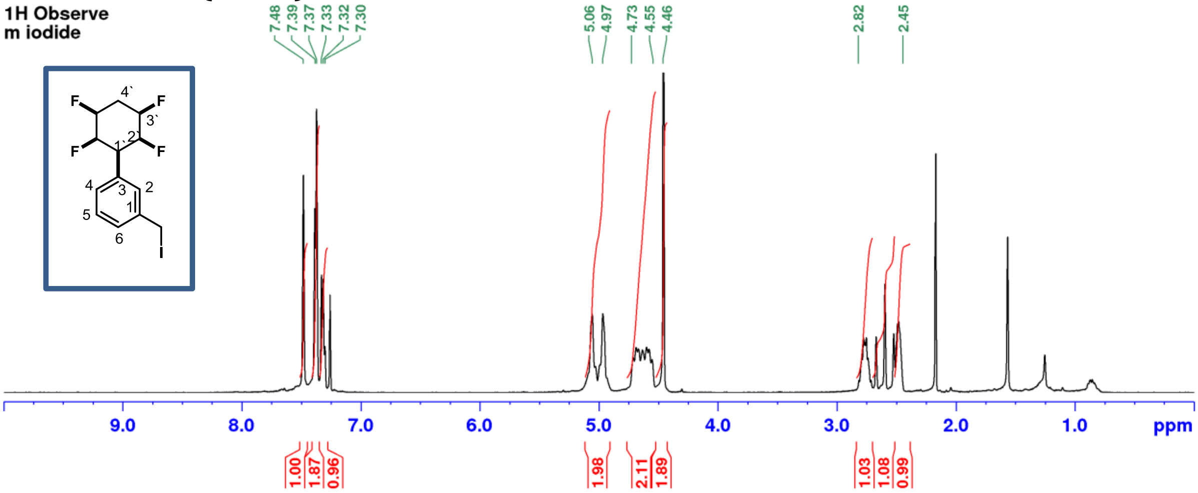

^{19}F{^{1}H} NMR of 29 ($CDCl_3$)

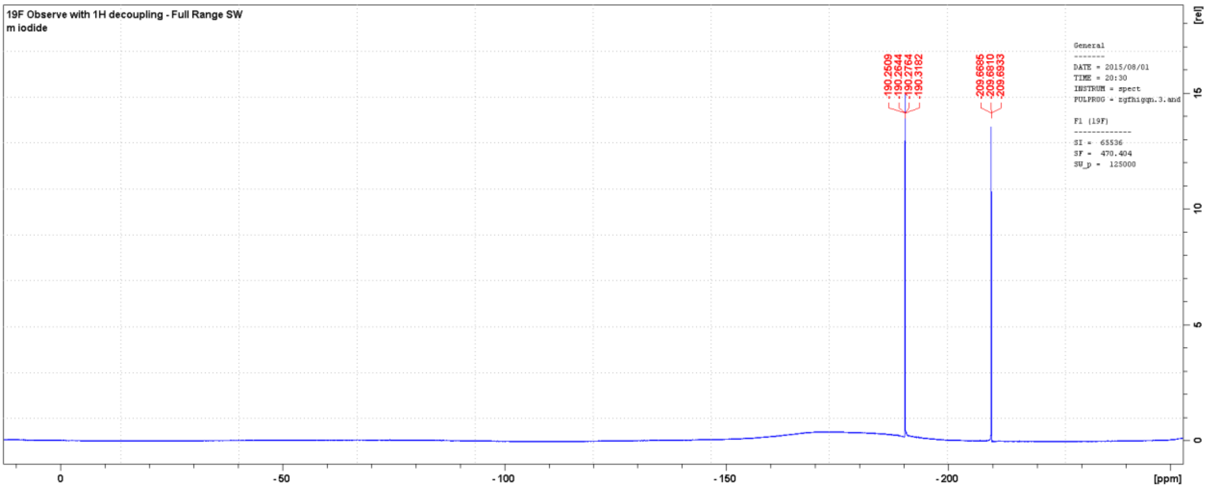

^{13}C NMR of 29 ($CDCl_3$)

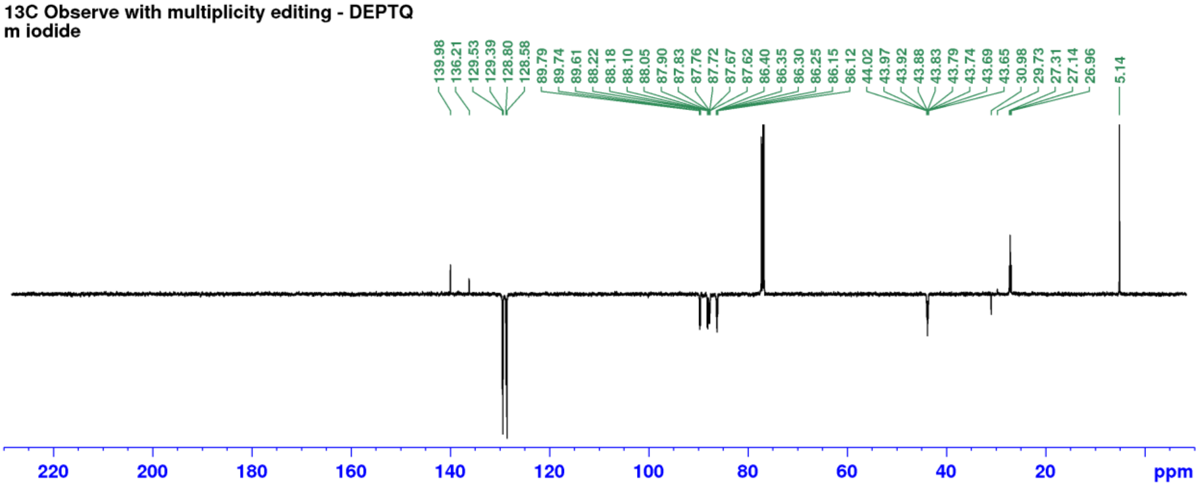

^{1}H NMR of 30 ($CDCl_3$)

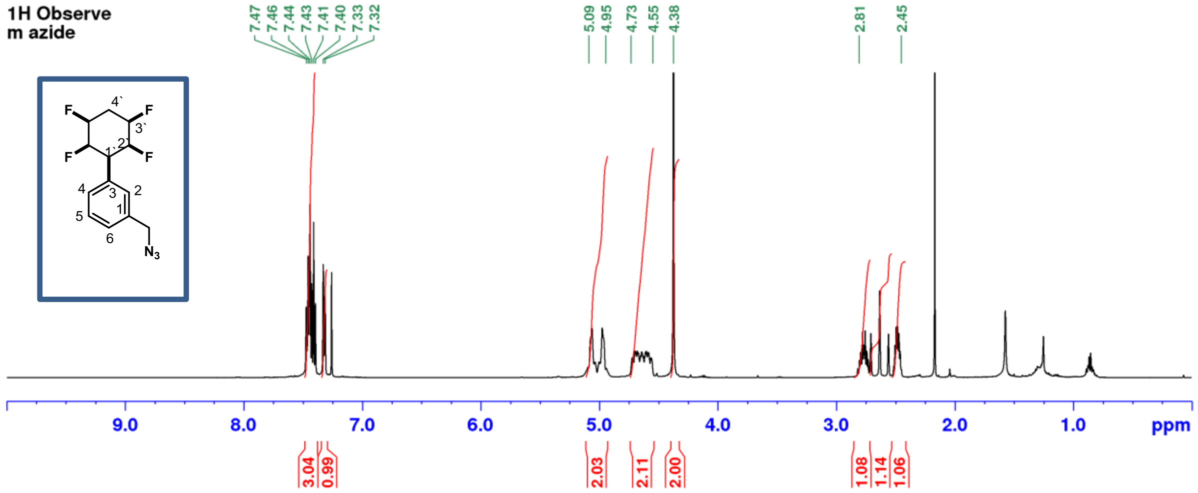

$^{19}F\{^{1}H\}$ NMR of 30 ($CDCl_3$)

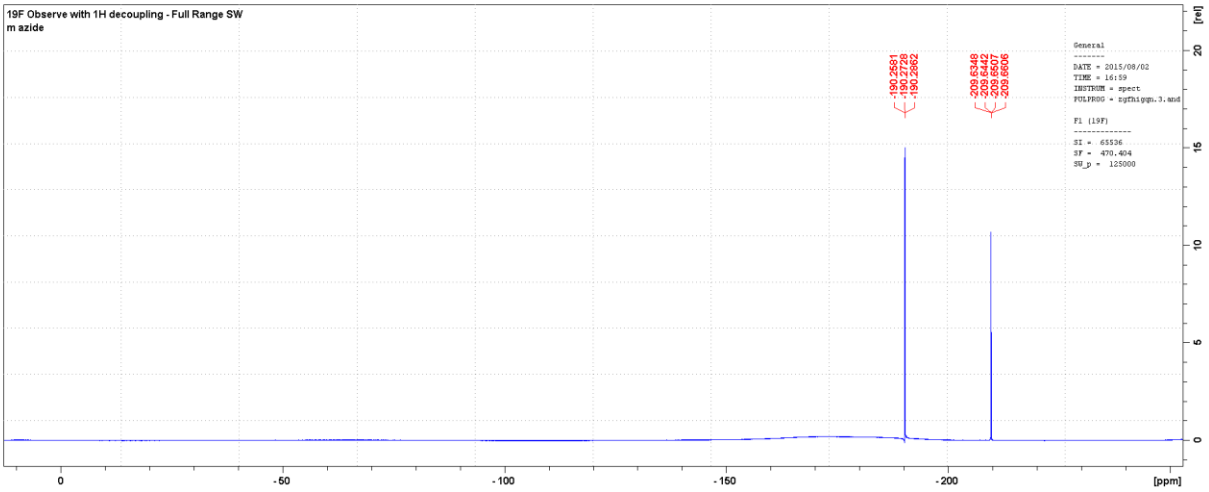

^{13}C NMR of 30 ($CDCl_3$)

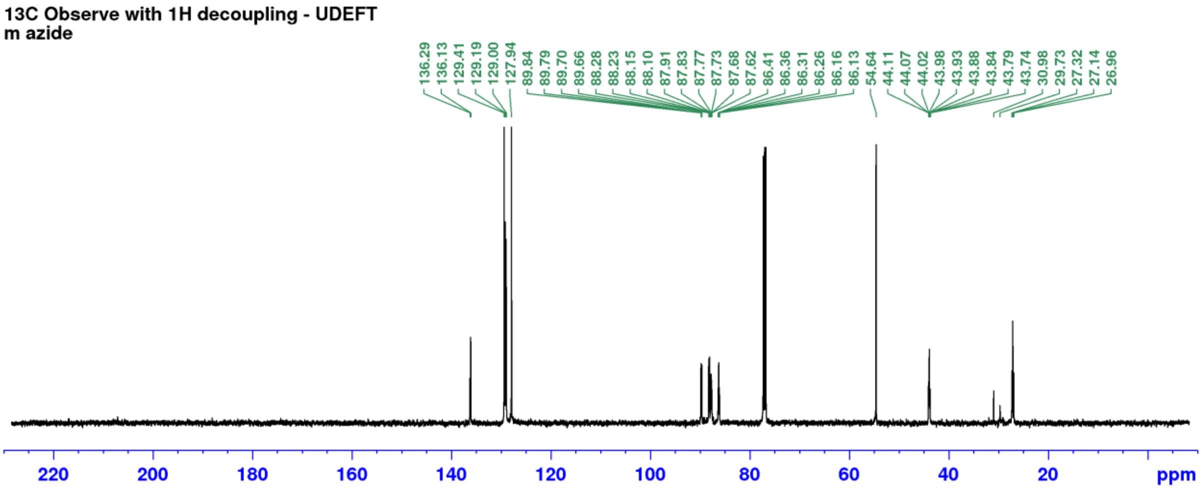

^{1}H NMR of 31 ($CDCl_3$)

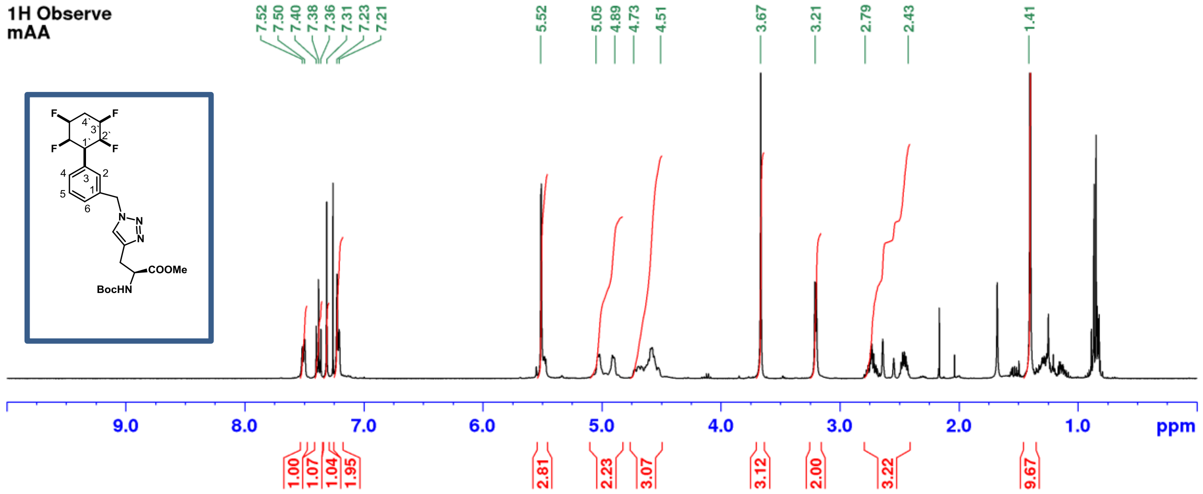

$^{19}F\{^{1}H\}$ NMR of 31 ($CDCl_3$)

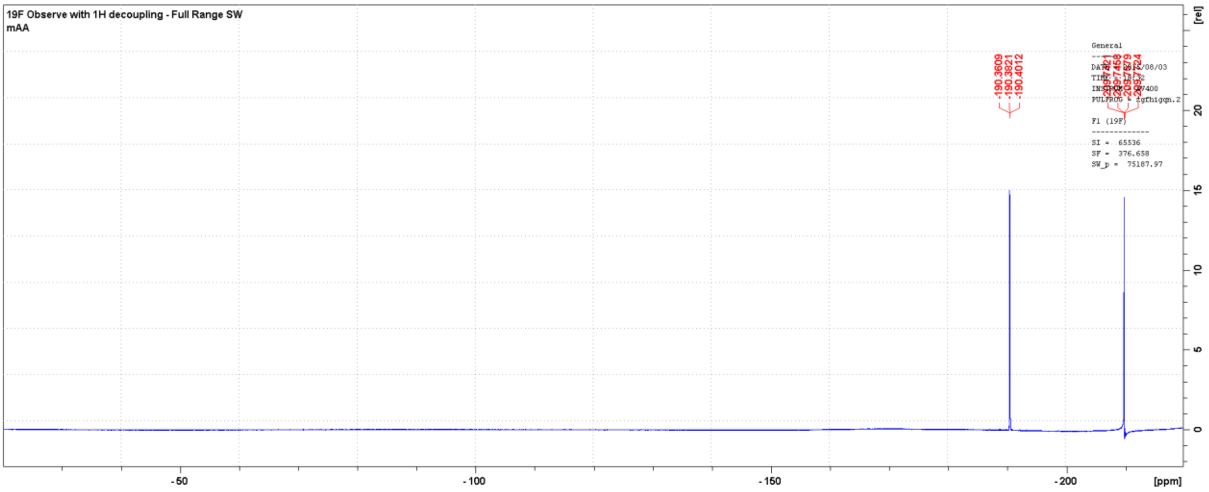

^{13}C NMR of 31 ($CDCl_3$)

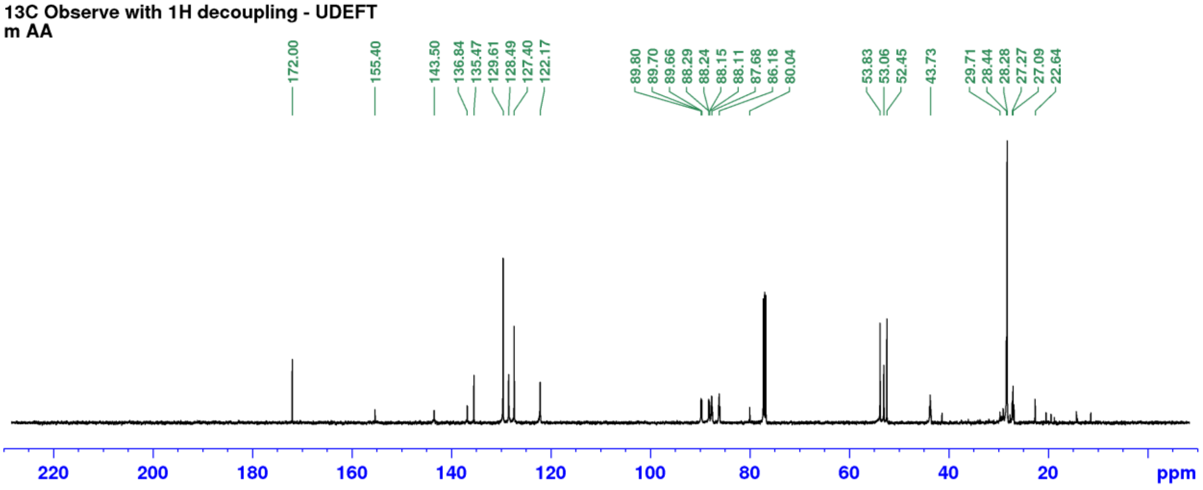

^{1}H NMR of 32 (d_6-DMSO)

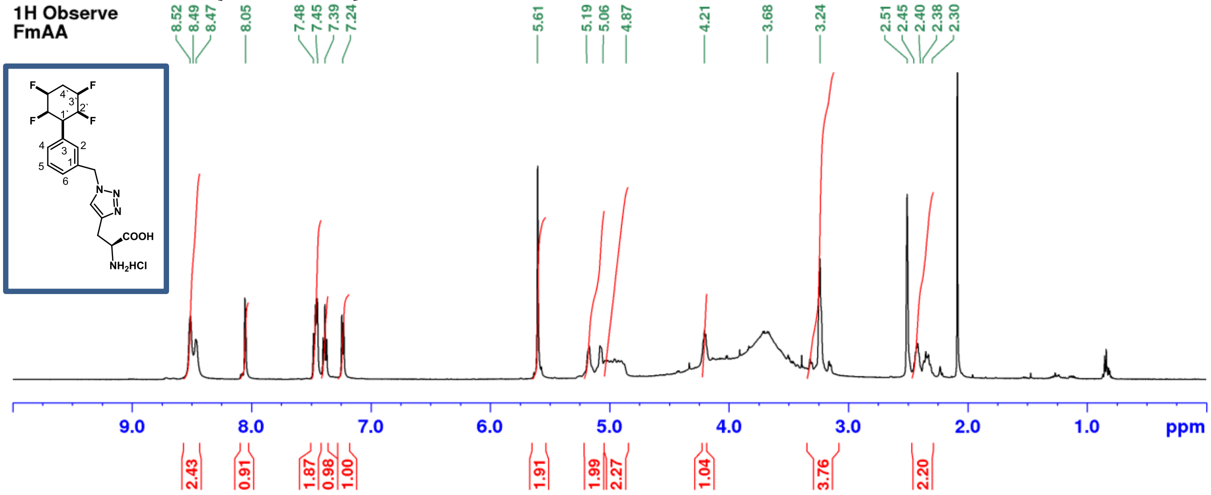

$^{19}F\{^{1}H\}$ NMR of 32 (d_6-DMSO)

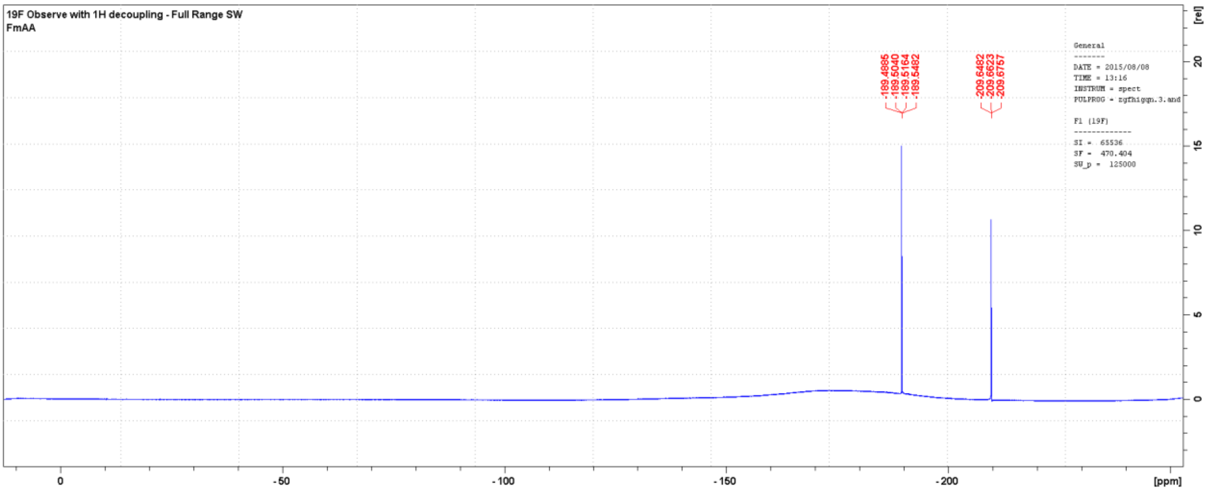

^{13}C NMR of 32 (d_6-DMSO)

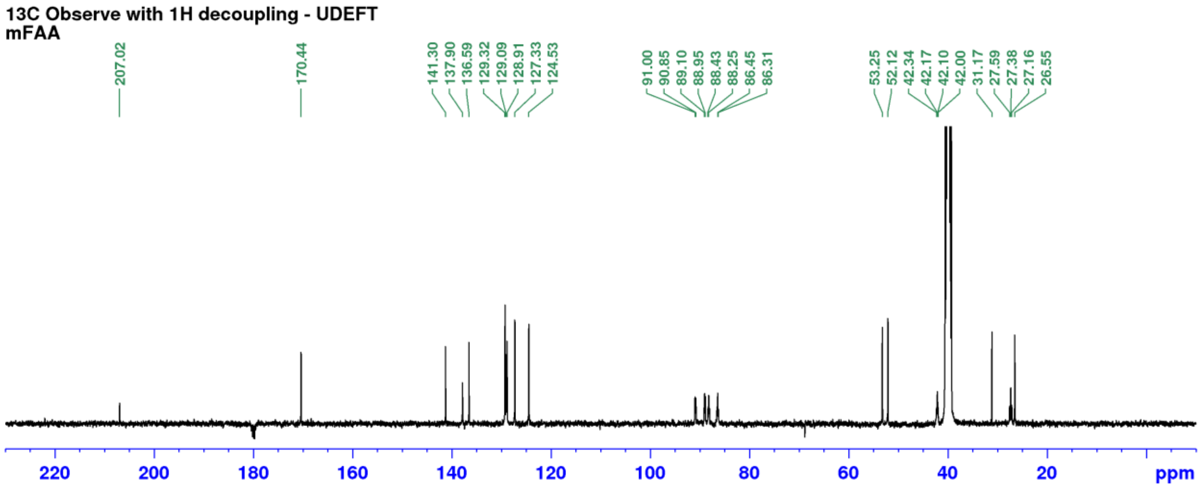

Supplement: File 1 — Experimental part. [file Beilstein_J_Org_Chem-11-2671-s001.pdf]
